# 日本のプライマリ・ケア医のコロナワクチン接種の実践――スケーラビリティとノンスケーラビリティのはざまで

**DOI:** 10.12688/f1000research.126366.1

**Published:** 2022-11-08

**Authors:** 木村周平 木村, 堀口佐知子 堀口, 後藤亮平 後藤, 飯田淳子 飯田, 小曽根早知子 小曽根, 金子惇 金子, 照山絢子 照山, 濱雄亮 濱, 春田淳志 春田, 宮地純一郎 宮地

**Affiliations:** 1 Faculty of Humanities and Social Sciences, University of Tsukuba, Tsukuba, Japan; 2 Temple University, Japan Campus, Tokyo, Japan; 3 Faculty of Medicine, University of Tsukuba, Tsukuba, Japan; 4 Faculty of Health and Welfare, Kawasaki University of Medical Welfare, Kurashiki, Japan; 5 Association of Medical Science, Yokohama City University, Yokohama, Japan; 6 Faculty of Library, Information and Media Science, University of Tsukuba, Tsukuba, Japan; 7 Tokyo College of Transport Studies, Tokyo, Japan; 8 School of Medicine, Keio University, Tokyo, Japan; 9 The Hokkaido Centre for Family Medicine, Sapporo, Japan

**Keywords:** COVID-19, primary care physician, Japan, vaccine, scalability

## Abstract

世界各国で新型コロナウイルス感染症への対策としてワクチン接種が進められている。本稿は2021年において、日本国内のプライマリ・ケア医がワクチン接種をどのように準備し、実施においてどのような問題に直面し、またそれにどのように対応したのかを、10人のプライマリ・ケア医への継続的なインタビューにもとづいて明らかにする。大規模で円滑・迅速に進めるべきワクチン接種プロジェクトは、チンのいう「スケーラビリティ」をもつことが前提されている。しかし実際には現場での様々な調整や情報共有、働きかけが必要であった。プライマリ・ケア医たちは、情報不足へのストレスや業務過多による疲弊を感じつつ、地域やかかりつけ患者への大事なサービスとして接種を行った。本稿の知見は今後のワクチン接種計画や実施の改善のための資料となるものである。

## Abstract (en)

Vaccine rollouts have been underway to combat the COVID-19 pandemic globally. Based on ongoing interviews with ten primary care physicians ‘in the field’, this paper elucidates how in practice the vaccinations were carried out in Japan in 2021. We examine what the primary care physicians did to prepare for the rollouts, what problems they faced, and how they responded to these problems. Large-scale vaccination projects are supposed to proceed smoothly and quickly, or to have what Anna Tsing calls ‘scalability’. In practice, however, they required a variety of tasks for coordination, information sharing, and promotion. Despite feeling stressed by the lack of information and exhausted by the work overload, the primary care physicians carried out the vaccinations as an important service to their patients and communities. The findings of this paper will provide valuable materials for improving future vaccine rollouts.

## I. 序論――ワクチン接種を記録する

本稿は、新型コロナウイルス感染症（以下、COVID-19）への対策として開始されたワクチン接種について、プライマリ・ケア医への継続的なインタビューにもとづき、(1) 日本では 2021 年にどのように行われ、プライマリ・ケア医の立場から見てどのような問題があり、かれらはそれにどのように対応したのかの実態を整理すること、(2) それによって、今後のパンデミックに向けての知見を提示することを目的とする。

COVID-19 を引き起こす SARS-CoV-2 に対しては、ワクチンの開発と接種がパンデミック終息の鍵のひとつと考えられ、かつてないスピードで進められた。ワクチンは結果として様々な国や地域において社会的な緊張の緩和に一定の役割を果たしたが、パンデミックの終息に至るまでの効果はもちえていない、というのが本稿執筆時点での現状である。

日本において、ワクチン接種は間違いなく2021 年の重要な出来事のひとつであった。政治家、行政、医療者、メディア、市民（反ワクチン派も含め）などが、それぞれの不安や期待を抱えながら、強い関心を持って、この問題に関与した。渦中においては少なからぬ混乱も生じたが、国を挙げてのプロジェクトとして見た場合、短期間で急速にワクチン接種率を高められたこと、（少なくとも部分的には）感染第 5 波の急速な減衰が起きたこと、またインフルエンザなど他のワクチン接種への警戒心も下がったことなどの点で「成功」と評価することも可能である。では、こうした混乱や「成功」はいかなるもので、どのように生み出されたのだろうか。

大きな出来事は、意外にも素早く忘却されることがある。文化人類学者の 早川 (2015) は、ジンバブエの人々が、年率 30 万%を超えた猛烈なインフレについて、数年後に「あれほど大変だったのにもうほとんど覚えていない」と語った、と記す。このことは、歴史家のクロスビーが 1918 年のインフルエンザ・パンデミックについての著作に『アメリカの忘れられたパンデミック』というタイトルをつけたこととも共鳴する ( Crosby, 2003)。今回のワクチン接種、さらにはパンデミック全体も、同じ道筋をたどりうる。実際、接種開始から時間が経ち、必要接種回数の増加や、新たな変異株の出現などで、「成功」のみならず、ワクチン接種の意義や評価も曖昧になりつつあるように見える。しかし、将来的なパンデミックの際に起きうる問題に備えるうえで、現場での対応に関わる記録を残すことには意義があるはずだと、私たちは考える (cf. 林・田中・重川, 2009； 木村ほか, 2020)。

以上の問題意識から、本稿では、ワクチン接種の実践を記述する。ワクチン接種というプロジェクトには政府や行政、製薬会社、医療者、市民（被接種者）など、多様な立場の人々が関与し、その立場ごとに異なる問題に直面していた。それらすべてをひとつの論考で取り上げることはきわめて難しい。そのため、本稿はこれまでの筆者らの調査に基づき、プライマリ・ケア医に焦点を当てて議論を進める。なお、プライマリ・ケアとは「国民のあらゆる健康上の問題、疾病に対し、総合的・継続的、そして全人的に対応する地域の保健医療福祉機能」（ 一般社団法人日本プライマリ・ケア連合学会ウェブサイトより）であり、それを担う医師のことをプライマリ・ケア医と呼ぶ。従来、「家庭医」や「かかりつけ医」と呼ばれてきた医師や、近年、19 番目の基本領域専門医として制度化された「総合診療医」などが含まれる。「全人的」などの語が示すように、プライマリ・ケア医のなかには患者の人格や個別性に配慮し、心理・社会的側面にも焦点を当てた丁寧な診療を志向する医師も少なくない。その意味で、通常は医師の中では後述する「スケーラビリティ」からは遠いところにいる存在であるという点が、かれらを対象とするうえで重要な点である。

以下、第 II 章では枠組みと方法を述べ、第 III 章でワクチン接種を概観する。第 IV 章でプライマリ・ケア医の実践について調査結果を提示し、それをもとに第V章で考察を提示する。

## II. 先行研究と方法――ワクチン接種という実践

### 1. 先行研究と枠組み

COVID-19 を含めた感染症に関しては、医学や公衆衛生学だけでなく、歴史学や心理学、哲学、文化人類学など、様々な学問において膨大な研究が蓄積されている。このうち本稿で扱うワクチンについて、筆者らの依拠する文化人類学を中心に研究を挙げれば、来るべきパンデミックへの「備え」のなかでワクチンについて触れたもの ( Caduff, 2015； Lakoff, 2017)、グローバルヘルスにおけるワクチンの問題を扱ったもの（ 浜田, 2017 など）、子どもへのワクチン接種に対する人々の受容、特にワクチン忌避や反ワクチンの動きを論じるもの（ Sabo, 2015； ラーソン, 2021 など）がある。そのなかで、ワクチン接種という実践に焦点を当てる研究はわずかである。確かに、1 回ごとの接種という行為は、医療に関わる行為の中で比較すれば高度な専門性が求められる技術だとはいえず ^1^、また時間としても 1 分足らずで終わってしまうため、「実践」として注目をひかないのかもしれない。

しかし、ワクチン接種自体が比較的容易な行為であるとしても、接種の増加による集団免疫がスムーズに実現できるわけではないことは、上で言及した「受容」や「備え」の研究が示している。「受容」の研究は、様々な社会集団（マイノリティを含む）が様々な理由からワクチン接種を忌避すること、人々を受容に導くには多様な方法でのコミュニケーションが必要であることを明らかにしている。また「備え」論は、ワクチン開発・接種が、（少なくともいくつかの国では）過去のパンデミックの経験を踏まえて、政府、医療機関、研究者、製薬会社、軍など多様な集団や人々が関わる、大規模で綿密な計画の下で行われるものであることを明らかにしている。当然、大規模なワクチン接種の実施では、様々な箇所において計画やシミュレーションでは予想していなかった問題が現れ、現場で即座の対応を迫られることになる。例えば Caduff (2015) は 1976 年の豚インフルエンザ流行危機下のアメリカでのワクチン接種キャンペーンにおいて、接種が進む中で副反応や、ワクチンに起因するとみられる重篤な問題が発生したことを指摘する。そのため市民からの反発が大きくなり、また流行が予想よりも小規模でとどまったこともあり、接種キャンペーンは途中でストップしてしまったという。

このように実際のワクチン接種が容易でないことを論じる上で、現代のガーナ南部における乳幼児のワクチン接種（3 種混合など）とイベルメクチン投薬のプロセスを調査した 浜田 (2017) の研究が参考になる。浜田はいかに地域保健看護師によって行われるそれらが、薬剤が要請するリズム（イベルメクチンであれば年 2 回）と、地域の人々の生活のリズムとの間を調整しながら進められているかを、薬剤の時空間への配置 ^2^という視点で分析した。そして、「薬剤の時空間への配置は非常に手間のかかるものであった。それは、人間の手足を用いて多大な労力をかけて行われ、また、人間の記憶力を補うための道具立てを必要とするものであった」（ 浜田, 2017: 644-5）と書いている。こうした「労力」はもちろん、地域によって、またワクチン自体の扱いやすさなどによっても大きく異なるだろうが、こうした「薬剤の配置」と「労力」に注目することは、ワクチン接種の実態を捉える上で重要になるはずである。

本稿ではこの点について、さらに チン (2019) が別の文脈で使った「スケーラビリティ」という語を導入したい。スケーラビリティは、例えば工業生産などの計画やプロジェクトの、小さいレベルで実現可能なものは単純に拡大すれば大きなレベルでも実現可能である（と想定される）性質のことである (cf. Scott, 1998)。逆にスケールを変えると性質が変わってしまう（実現できなくなる）もののことを、チンは「ノンスケーラブル」だと言う。

このスケーラブル/ノンスケーラブルという語を使うと、パンデミックへの対応としてのワクチン接種プロジェクトは、ミクロ（ウイルスに対し効果を発揮するワクチンの開発）から個別のワクチン接種の実施、そして地域、国家、世界というスケールでの集団免疫までが、スケーラブルであるという前提をもつと考えることができるだろう。しかし、先程の「薬剤の配置」と「労力」に目を向けるなら、そのスケーラビリティは、あくまでも前提であり、実際には、様々な主体が、プロジェクトをスケーラブル「にする」ために、自分の可能な範囲で問題を解決したり回避したりという諸実践を行っていると想定できる。

以上の検討をふまえ、本稿では「ワクチン接種」をスケーラビリティという観点から捉え、特に日本のプライマリ・ケア医に焦点を当て、「誰かの身体にワクチンを接種する」だけにとどまらない幅広い――上でも述べたように、通常時とは異なる志向性のものも含んだ――実践について、かれら自身の言葉をもとに整理し、考察する。

### 2. 方法

筆者らは、パンデミックが日本で顕在化してきた 2020 年 3 月より、国内各地で勤務する 10 人のプライマリ・ケア医（ 表 1）に対して、2~3 か月に 1 回程度のオンライン・インタビューを継続してきた（ 飯田ほか, 2021； Haruta *et al.,* 2021； 木村ほか, 2022）。本研究については、日本プライマリ・ケア連合学会の倫理審査を受け、承認されている（承認番号 2019-013）。日本プライマリ・ケア連合学会の倫理承認を得た理由としては、当学会が認定された学会であり、倫理承認を得るのに適切な場であると参加者と著者に認識されていたこと、研究チームの半数が当学会に所属していたことが挙げられる。

**表 1.** 調査対象者

略号	A	B	C	D	E	F	G	H	I	J
ｼﾞｪﾝﾀﾞｰ	M	M	M	M	M	W	W	M	M	M
地域	北海道	関東	首都圏	首都圏	首都圏	中部	中部	関西	中国	九州
	周辺部	都市部	都市部	都市部	都市部	周辺部	周辺部	都市部	都市部	都市部
勤務 施設	診療所 (公立)	病院	病院・診療所	病院	診療所 (私立)	診療所 (公立)	診療所 (私立)	診療所 (私立)	診療所 (私立)	病院・診療所
1 回目	3/23	2/3	2/8	2/3	2/23	2/8	2/2	2/2	3/23	3/12
2 回目	6/21	4/16	4/30	4/16	5/22	4/30	5/8	5/8	6/21	5/21
3 回目	9/21	8/2	7/19	8/2	8/18	7/19	8/15	8/15	9/21	9/3
4 回目	11/30	11/18	11/20	11/18	11/21	11/22	11/30	11/30	11/30	11/18

研究方法については、 照山ほか (2021) で述べているが、COREQ のチェックリスト ( Tong, Sainsbury and Craig, 2007) をもとに、以下に記載する。対象となる 10 人を地域や勤務機関、ジェンダー等を考慮して選定した。対象者は研究チームのうちの医療者のネットワークを通して依頼したため、調査開始以前にチームの少なくとも 1 人とは面識があったが、そのことがインタビュイーを信頼して経験や感情を比較的自由に語ることにつながった。調査開始にあたり、対象者個々に研究計画を説明し、同意書に署名してもらった。なお、10 人全員が調査への参加に同意し、拒否した人はいない。インタビューは、基本的にはインタビュイー 2 人に対し調査者 4 人（医療者 2 名と人類学者 2 名、いずれも質的調査の十分な経験がある。また、インタビューでは毎回対象者と同じジェンダーの調査者が同席する）というグループで行った。質問は、人類学の方法にもとづき、構造化しておらず（質問票は使っていない）、新型コロナウイルス感染症への対応や、現場から見える地域の様子、そしてその時々の話題（例えば 2021 年にはオリンピックやワクチン、変異株など）について、できるだけ自由に語ってもらうという形式をとった。インタビューは毎回 1~2 時間程度であり、人類学者のメンバーの一人がメモを取りつつ、録画・録音した。

本稿では、これまでに行った計 75 回のインタビューのうち、2021 年 2 月から 11 月までに行った計 25 回のインタビューをデータとして用いた。まず、録画・録音したインタビューを文字起こしし、そこからワクチンに関わる部分を抽出し、内容のまとまりごとに分類した（文化人類学の手法に基づいているため、厳密なコーディングを行っているわけではない）。その整理したものをもとに、時系列に沿って大きな動きを把握するとともに、傾向やパターン、および個々の特異性を分析するという作業を行った。第IV章以下では、そのようにして見いだされたパターンや特異性に合わせて、それをよく示す語りを提示している ^3^。そのうえで、そうしたパターンや特異性を生み出す背景について考察した。

なお、本稿執筆にあたっては、執筆者全員でデータ収集と整理を行った。そこから木村を中心に分析と草稿執筆を進め、草稿について全員で議論し、堀口・後藤・飯田を中心に加筆修正提案をまとめ、木村を中心に提出稿を作成した。

## III. 背景――ワクチン接種の経緯とスキーム

### 1. ワクチンの開発と接種

本章では、次章で取り上げる具体的な事例のための背景として、2020 年から 2021 年の間の、ワクチン開発から接種にいたる大きな経緯、および日本国内での接種のスキームを概観する。

新型コロナウイルスが発生したとされるのは 2019 年の年末であったが、ワクチン開発にとって基礎となるウイルスのゲノム配列は 2020 年 1 月の段階ですでに解析・公開されている ( Wu *et al*., 2020)。WHO や有力な財団などによる国際的連携への働きかけもあり、ワクチン開発は通常ないほどに大規模かつ速いスピードで進められた。他方で、複数回感染の症例が報告されるなど ( To *et al.*, 2020)、ワクチンの効果に対して悲観的な見解もあった。しかし、2020 年夏以降、ロシアや中国、イスラエル、イギリスなどの政府がそれぞれ、開発されたワクチンのいずれかを承認し、接種を開始した。他の国々もそれを追うように接種に向かった。日本政府は 2020 年夏に複数の製薬会社と数千万回分の供給契約を結び ^4^、12 月にワクチン接種を無料にするために予防接種法を改正、また同月に最初の「新型コロナウイルス感染症に係る予防接種の実施に関する手引きについて」を公開し ^5^、月に 1 回程度（2021 年夏以降は 2 か月に 1 回程度）、自治体向けの説明会をオンラインで行うなど ^6^、ワクチン接種を円滑に行う準備を進めていたが、ワクチンの薬事承認は他国から遅れをとり、2021 年の 2 月に入ってからのことであった ^7^。

承認後、日本政府はまず 2 月下旬より医療者、次いで 5 月より市民に（重症化のリスクの高さを考慮して、高齢の人から順に）、という接種方針を示した ^8^。医療者への接種は都道府県、市民へは基礎自治体と担当を分けて進めることになった ^9^。12 月から承認を受けたワクチンは mRNA ワクチンという、従来とは異なるタイプのものであり、そのための扱いの難しさや、副反応の激しさ、長期的なリスクが明らかでないという懸念による接種控え、さらには製薬会社からの供給量が安定しなかったことなど様々な混乱が生じ、計画は予定より遅れた。次章で見るように、こうした混乱を乗り越えてワクチン接種を円滑・迅速に進めるために、「スケーラブルにする」諸実践が行われた。

4 月半ば、感染第 4 波（主にアルファ株）が大阪を中心に拡大し、ワクチンの遅れへの批判が高まると、菅義偉首相は 4 月 23 日に高齢者への接種を 7 月末までに完了すること ^10^、5 月 7 日には 1 日 100 万回接種すること ^11^を宣言、さらに渡米して製薬会社と交渉し、供給量を増やす約束を取り付けた。さらに河野太郎担当大臣は大規模接種や職域接種などの実施も打ち出した。政府方針の突然の発表や変更によって現場はさらに混乱しつつも、接種は強力に推し進められていった。

その一方で感染者数は再び増加し（デルタ株を中心とする感染第 5 波）、オリンピック開催直前の 7 月 11 日には東京都に緊急事態宣言が発出された。ここでの重症者や自宅療養者の急増は、大規模なワクチン接種の後押しともなった。その一方で、ワクチンへの異物混入事件や、地域によってはワクチン供給量が不安定になるという事態も起きた。

第 5 波は 9 月に入ると急激に減退していき、10 月から 12 月まではきわめて感染者数の少ない時期を迎えた。ワクチン接種率は 10 月半ばの時点で 65% を超え ^12^、ワクチン接種の「成功」を称える声も出た。しかし、12 月に始まる第 3 回目のブースター接種の接種率が伸び悩み、さらに感染第 6 波が始まり、ワクチンの効果が弱い（が重症化しにくい）とされたオミクロン株が広まるなかで、2021 年のワクチン接種を振り返る議論は聞こえなくなっていった。

### 2. ワクチン接種のスキーム

次にワクチン接種のスキームを説明する。新型コロナウイルス感染症対応は 2009 年の新型インフルエンザ流行などを契機に策定された法規や計画をもとに進められ、具体的なワクチン接種の進め方については国が示す「手引き」に依拠して行われた。ただ、都道府県や自治体、医療機関等の裁量に任された部分も少なくなく、各レベルで接種をより円滑に進めるためのローカルな調整が行われており、多様性が生じている。この点でもスケーラブルだという前提と現実とのズレが見出せるのだが、ここではあくまで説明のため概略について述べる。

ごくシンプルに考えれば、ワクチン接種には「接種する者」と「接種される者」「接種するワクチン」および「接種場所」の 4 つの要素が必要だといえるだろう。以下、順にそれらを見ていく。

まず「接種する者」だが、誰が接種するかは、接種に関わる法規や、接種という行為の難易度やリスク、および「どれほどの人数にどれほどのスピードで接種するか」ということの関数でもある。今回は大量の接種を迅速に行うため、医師や歯科医師のほか、看護師や保健師、助産師なども接種者となり、各「接種場所」での接種を行った。各「接種場所」では、実際の接種者のほかに、その場所での接種の管理・運営にあたるため、多くの人が関わった。

次に「接種される者」は、広くは「日本に在住する人々」とされ、その全体の接種率を上げることが目指された。そのため、社会に対し適切な情報を提供しつつ、順序よく接種へ誘導すること、そして副反応による被害を抑制すること、などが課題となった。

そして「接種するワクチン」だが、前節でもふれた通り、今回の mRNA ワクチンは取り扱いの難しいものであった。具体的には、脆弱なために冷蔵での輸送やディープフリーザーでの保管が必要であること、バイアルと呼ばれる容器に複数回（5 回ないし 6 回、この容器から注射器に移し替える作業も神経を使う）の接種が可能な量が入っているが、解凍したバイアルは一定の時間内に使い切る必要があること、さらに数週間の間隔をあけて 2 回接種が必要であること、さらに加えてワクチンを無駄にしたり、廃棄したりしないようにという大臣発言によるプレッシャーがかかっていたこと、などが挙げられる。こうした問題は行政や接種機関を悩ませた。

「接種場所」は、行政が医師会などと連携して指定した。接種を希望する医療機関（病院・診療所）のほか、行政が設置する大規模接種会場、さらに職域接種の場合は職場指定の会場であり、それに加えて高齢者福祉施設等での集団接種や、移動の困難な市民に対する在宅での接種も行われた。詳しくは後述するが、各医療機関のうち、接種後の待機場所の必要性、通常診療や経営面への圧迫などから、接種をしない選択をするところもあった。他方、医療機関の少ない地域ではその選択は難しかった。

これらに加えて、今回のような大規模のワクチン接種においては、この 4 つをうまく調整し「配置」する（ 浜田, 2017）ことが必要になるが、その役割は主に行政が担うこととなった。前節で述べたように、医療者を除く市民については重症化リスクの高い高齢者からという国の方針に従い、自治体はおおむね、年齢でグループ分けして接種券を発行・郵送し、「接種場所」と連携しながら予約・予診・接種の実施や接種記録の管理を行う仕組みを作った。誰にいつ接種券を配布するかというグループ分けやタイミング、さらに予約などの詳細も各自治体に任された。そのため自治体は、ワクチン担当の部局を決め、医師会や専門家委員会などと連携しつつ、「接種場所」を確保し、全体のワクチン供給量の推移も見ながら、市民がどのぐらいの人数、どこでの接種を予約するかを想定し、混乱のないよう接種券の発送や予約、ワクチンの供給などを行おうとした。独自のやり方で成功した自治体もある ^13^。しかし、ワクチンの取り扱いの難しさや供給の不安定さ、同時に進んでいた都道府県による医療者接種とのバッティング、担当職員の過大な業務負担などの様々な要因により、遅れを取る自治体もあった。各医療機関や医師たちは、そうした自治体ごとのやり方や、その差異によって大きく翻弄されることとなった。

次章ではその実際の様子を、プライマリ・ケア医の語りを通して明らかにする。

## IV. 結果――プライマリ・ケア医の実践

### 1. 見通しの悪さのなかでの接種

ワクチン接種に向けてプライマリ・ケア医たちが動き始めたのは、国内での認可など接種が現実化してきた、そしてちょうど感染第 3 波も落ち着いてきた 2021 年 1~3 月頃であった。

本調査のインタビュイーたちの実践は、大まかには①自治体の方針策定に関わり、自身でも接種を行った医師 (A、E、F、G)、②自治体の方針のもと、接種に携わった医師 (C、H、I)、③ワクチン接種の動きにはサポートする立場に立った医師 (B、D、J)、の 3 つに分類できた。ここからは、プライマリ・ケア医たちのワクチンに関わる実践は、必ずしも接種そのものだけではない、ということが明らかになる。大まかには①は、普段から自治体や医師会との距離が近かった医師、③は病院勤務で新型コロナウイルス患者の診療にあたった医師であり、②はそれ以外の医師だといえるが、おそらくプライマリ・ケア医を全体的にみれば、大多数の医師は②に含まれるだろう。

まず、①の医師の関わった業務は、具体的には、接種方法等の話し合い、医師会員向けへの情報提供、集合契約の連絡、とりまとめ、行政と予約方法や接種場所（個別，集団）やそのレイアウトの検討 (G 0202)、さらには誰に優先的に接種するかの判断 (G 0815) など、多岐にわたる。これらの医師は、②、③の医師と比べて当然仕事量が多いが、情報へのアクセスがよく、程度の差はありつつも、以下の語りのように、ワクチン供給に関わる国の動きや、各医療機関への配送スケジュールなどについて知ることができたようである。

2 週間ごとにクールがあって、それによって自治体に何箱配送する、ということが決まっているのですけれど、やっと 6 月末までの配送が今、各自治体の数が決まってきたので、ワクチンの数が決まると、どういう接種スケジュールが現場で作れるか、という話になる (G 0508)

こうした情報は当然、自身の医療機関での接種計画に役立てることができる。しかしだからと言って計画と実施がスムーズだったわけではない。①の医師の語りには、国の方針の変更によって行政が受けた大きな影響も述べられている。

最近すごく大変だったのが、数日前に河野大臣が高齢者は 7 月いっぱいで終わらせると (…) おっしゃったではないですか。(…) 高齢者の接種出来る期間を私たちは 8 月くらいまでは見積もっていたのですよ。だから、一気に接種の期間が 3 分の 2 にガンと減ったので、もう2000 人近い人を一気に集団接種で前倒ししなければいけなくて、それで各医療期間で接種出来る数も増やさなければいけないし、集団接種で 1 回 200 人とか予定していたのを、350 人くらいにしなければいけなくなったりして、そのリスケジュールがすごく大変だった (G 0508)

このように、より上位レベルの急な方針の提示や転換は、より下位のレベルでの決定や実施を振り回すことになった。この点は、②の医師たちの語りに顕著に見られる。かれらは自治体からいつ・どのぐらいワクチン供給がなされるか（接種を実施するのか）に関する情報がなかなかはっきりせず、急に決まったり、変更されたりすることに翻弄されていた。

この 2 週間くらいから、急にワクチンのことの対応がどんどん決まって、何かずっと情報が分かっていない、隠されたような感じで、やっと 1 週間前から毎日ホームページで少しずつ、少しずつ更新されて (…) 何時と何時にどこで打てるということがやっと分かっているような感じですので、その辺はかなり診療所自身も混乱しているというところがあるなと思います。(C 0430)

個別接種でどれくらいの頻度で、何本配達しますか、みたいなアンケートも、4 月 28 日の水曜日の夜中に来ていて、29日が祝日で、30 日にみんな見て、締め切りが〔5 月〕1 日だったのですよね。(…) それがまた FAX で返事だったの〔ですが、先方は〕ずっと話し中で誰も送れなくて、電話も繋がらなくて、各クリニックもすごくパニックになっていました。(H 0508)

この 3 か月はワクチンのことが一番大きかった。こんなに行政のホームページを毎日にらめっこってなかった。〇〇ワクチン最新情報がどんどん更新されていく、それに追いつきつつ自分たちの動きを変える、それが大変。(C 0719)

このように、とくに②のプライマリ・ケア医の立場から見ると、ワクチン接種は全くもってスケーラビリティをもつプロジェクトなのではなく、供給されるワクチンと接種するべき人々とを、日々何とか調整しながら進めるというものだった、ということができる。ただし、①と②の対応や見通しの差異を見ると、行政や医師会との間のコミュニケーションの円滑化（情報提示やよりリアルタイムでの情報を提示できるツールの利用など）によって、多少状況を改善させられる可能性も指摘できる。

### 2. 接種にむけて働きかける

前章で述べた通り、医療者の 2 回の接種は 3 月頃から本格化し、市民の接種はそれと重なるように 5 月頃から始まり、秋頃まで続いた。

そのプロセスにおいて、かれらはワクチン接種の効果についてつねに確信できていたわけではない。初期にも「この現状を打破するのはワクチンしかないと思ってる」(D 0203) のような意見と、「〔他国の状況も見ると〕実際、やっぱり少なくとも 1 年とかは全然ワクチンの効果を実感することはないんじゃないかなと思うんですよね」(J 0312) という意見は両方あり、また終盤にも「日本のマスクとワクチンさえ、上手く行けば…。(…) 前よりは、少し楽観的になってしまっている」(I 1130) と「それ程、明るい未来は描いていなくて、(…) ずっとワクチン開発と変異株の登場のいたちごっこが、(…) 10 年とか 20 年とか続いてしまうのだろうかとかと想像している」(A 1130) という意見があった。また、同じインタビュイーでも、時期によって変化が見られた。

しかし、そうした揺らぎの中でもかれらはワクチン接種を続けた。少なくとも本研究で被調査者となったプライマリ・ケア医たちに関しては、ワクチン接種すること、および接種率を高めるという目的についての疑いの言葉は聞かれなかった ^14^。この点は今回のインタビュイーの偏りと言えるかもしれず、改めて検討が必要である。

接種に向けたプライマリ・ケア医たちの実践には、接種そのものだけでなく、接種を円滑に進めるための働きかけも含まれている。これは本稿の枠組みでは「スケーラブルにする」働きかけだと考えることができる。具体的には (1) 医療機関内や外部での勉強会、(2) 市民・患者への案内や呼びかけ、(3) 個別の対応、である。

とくに初期には、医療者のなかでもワクチンに対する警戒、懸念が大きかった。そのため、勉強会、研修会などを行い、ワクチン接種を円滑に進めるための情報提供を積極的に行っている（これは①、②、③すべてのグループの医師について言える）。ある医師は「院内で意識調査をしたけれど、3 分の 2 ぐらいワクチンを打ちたくないと言っている。なんとなく副作用が怖いとか。研修医とかは「アジア人のデータが少ない」とか。わかってること、わかってないこと、打つことのメリットデメリットは説明しないといけないなと思って」(D 0203) 勉強会を開催し、接種希望が 90% 近くまで増えたと語る (D 0416)。また①の医師の中には、様々な組織をつなぐ役割を果たしたものもいた。

市の医師会向けに研修会を、オンライン研修会を 2 回やったのですね。(…) 医師会だけでなくて、歯科医師会と薬剤師会の先生にも(…) 講演を聞いていただいて、3 師会合同で〔集団接種を〕やりましょう、という感じで、ちょっと機運を盛り上げたりしたり、とかですね。後は、(…) 医療機関で働いている医療従事者、看護師とか事務員向けにオンラインで講演会を、(…) 1 時 30 分とか、昼休みの時間に 1 時間くらいやって、ワクチンの説明をしたり、もう一つ最近は、市のワクチンの推進室の人に出席していただいて、いろいろと接種券の扱い方だとか、ワクチンの運搬をどうするかとか、そういう実務的なことを最近、先週くらいに、その講演会をやったりして、なるべく情報を常にお伝えしていって、というふうなことをやっていました。(G 0508)

市民・患者からも、早い段階からワクチンに関わる質問が出始めていた。そうした質問に向けてお知らせを出したり、応答したりということも行っている。

〇〇市の広報とかを使ったりして、市民向けに情報を流したりだとか、市のホームページで解説をして流したりだとか、後、市のワクチンのホームページを作っているのですけれど、そこで情報を流したりとか、という感じで、なるべくとにかく情報を流していく、正確な情報を流していく、ということをまず一つ頑張った。(G 0508)

診療所の全戸配布の瓦版でワクチンに関する (…) 住民向けの情報をお伝えするっていうことをやっていて。同時に診察室で、もう直接作ったリーフレットを手渡ししながら、ワクチンに関する情報をお伝えしている。(A 0323)

それに加えて、予約時期が近づくと、予約の仕方など具体的なことに関する市民からの問い合わせも増えてくるため、それに対する対応も行っている。

電話ではどんなことが聞かれるから、これを用意しておくとか、何か予約番号と、後は、希望の場所と日時を指定しなければいけなくて、希望する時間とか場所とかをどういう風に決めたら良いかとかを、診療所の壁と、後、ゴールデンウィークだと診療所は閉まっているので、でも 5 月 6 日から予約が始まるので、外にその説明を全て貼って、診療所が閉まっていても見られるようにしてきました。(C 0430)

こうした情報提供は本来、自治体の役割だといえるが、普段から接している医療者に直接的に聞こうとする人々も多かった。医師たちは、主には小規模な自治体の場合 (A、F) は地域に向けて、大規模で他にも医療機関のある自治体では普段診療している患者に向けて、情報提供をしていた。

接種の予約は、自治体ごとに作った予約システムを使うところもあれば、直接医療機関で予約できるところもあった。自治体の予約システムの場合も、予定よりも多くの予約を受け付けてしまったり、システムと各医療機関の予約の連携が難しかったりと、しばしばトラブルが起き、医師たちもその影響を受けた ^15^。直接予約の場合はとりわけ医療機関への負担となった。問い合わせの電話の多さは相当なもので、さらになかなか予約できない状況になると怒ってクレームを入れてくる人もいた。医師たち自身がそうした電話に出ていたわけではないが、スタッフの疲弊や、電話対応専門にスタッフを確保したことについての語りもあった (I 0621)。

それらに加えて、夏にかけて、予告なくワクチンの供給量が減る事態もあり、すでに入っていた予約を断るため、患者一人一人に電話して謝り、いつに変更できそうか供給予測を医師会に電話して聞いて、また本人に電話する、というやりとりを行うこともあった (C 0719)。

市民とのやり取りを通じて、プライマリ・ケア医たちは市民の側のワクチン受容や、その変化も感じ取っていた。語りにはそうした傾向・変化やその背景についての解釈も含まれていた。プライマリ・ケア医たちの語りでは、4 月前半は接種を躊躇する人々の指摘が多かった。この躊躇の背景として、何人かはメディアの影響を強調した。

〔不安の源は〕もう本当にテレビだと思います。本当ずっと家に居るので、ずっとテレビを、ワイドショーを見てて、あれでもうやっぱり危ない危ないみたいな感じのことをいったり、専門家によっていうことがバラバラなので。(I 0323)

しかし、4 月後半以降には、積極的に希望する人の増加を指摘する語りが増えていった。

最近、この 1、2 週間からはいつ打てるのかという人がどんどんどんどん増えているというか、そういう人ばかりになってきたなという風に、みんなの流れが変わってきたなと感じています (C 0430)

こうした変化の背景として、かれらが語る日本人論や世代論も興味深い。プライマリ・ケア医たちは、診療などで接した具体的な人々を通して、より大きな対象集団のイメージを形成し、それをまた診療や患者とのコミュニケーションに生かしている（ 木村ほか, 2022）。

日本人的な考え方というのは結構あって、皆が打つのであれば〔自分も〕打った方が良いのではないかと〔考えたり〕、後は打ちにくさ、電話のかかりにくさとか、いつ打てるか分からない感じが出てくると皆打ちたくなる(…)。皆打ちたくて、注射がなくて、順番待ちだ、みたいになると、急に皆、電話をかけまくるみたいな。(F 0430)

高齢者は、たぶん僕が思うに、迷惑を掛けたくないから打っているっていうイメージがあるんですよ。自分がなるのも怖いけど、なって入院したり、それで葬式に来れないみたいなのになって、周りに迷惑掛けたくないから、周りの人のために打つみたいなのもあって、接種率が高いんじゃないかなというふうに予想していて。(I 0621)

周りを見て、打っている。最初に先頭を切って打とうと思わない方々、やはり周りが打ち始めていて、打った人の声を聞いて、結構、大丈夫そうだというのを見ていた人たちが、打ち始めたので、接種率も上がったのかなと思います。(J 1118)

プライマリ・ケア医たちが社会や集団にこのようなイメージをもっている一方で、かれらの個に対する見方もまた注目に値する。かれらは基本的には本節で見てきたように、集団に対しては接種を進める姿勢をとっているが、それでも「接種をしない」という判断をする人々については、個別に考え、深追いしない態度をとっているのである。

1 回目打たないと選択した方もいました。(…) 妊娠中の方々とか、後はポリシー的な部分でしょうか、医者の中にもいましたよ (…) それはある意味自由かなとは思っていて、あまり強制しても仕方がないところもあったので、変な話、一緒に〔コロナ陽性患者を〕診ている内科医の中にも打たないという人はいたので、それはそれでという感じでしたけれど。(B 0416)

医療施設においては接種するよう同調圧力が働いていたことを示唆する語りもあったが ^16^、しかし接種しないという意思を明らかにするスタッフについては、その選択が容認された。このように、可能な集団には積極的に働きかけ、説得の困難な個は深追いしない、というプラクティカルな態度が取られていた。

病棟のナースから〔反ワクチン派になってしまった親族について〕相談されることもあるのですが(…)、何を言っても、多分、それを信じてしまっている人には、多分、何を言っても届かないので、なかなか、修正、無理やり修正しようとしたら、軋轢が生じるだけだから、少し様子を見るしかないのではないかと言っています (…) その人たちが悪いと言っているわけではないのですが、その様に信じてしまっている人たちも、それを無理矢理修正するというのは、何か時が来ないと難しいと思って。(D 1118)

こうした姿勢は、プライマリ・ケア医たち自身を守るためのものでもあったのかもしれない。というのも、身近な反ワクチン派の人々の存在は、かれらを消耗させたからである。

日々コロナの診療をして、ワクチンを打ちに行っていて、「ワクチンなんて効かないし、害だから打つな」みたいな人達がいるみたいなことを、目の当たりにして、これ程、疲れることがあるかと言う (…)。フェイスブックとかを見ると、ワクチンみたいなものは、絶対に打たないみたいなものが、結構、出てきたりして。(…)しんどいので、結構、見ないようにして。(F 1122)

実際にどこまで働きかけ、どこからは深追いしないかの線引きは、医師ごとに異なり、ある個が周囲の判断に影響を与えうることを重視し、働きかけを続ける医師もいた。例えば、ワクチン接種年齢が下がる中で、学校の教員からの抵抗を感じた医師は、かつて HPV ワクチンを望む生徒が養護の教員に接種を止められるということを経験していたため、自治体の校長会で講演し、「恐れることはないので、子供たちにも、集団生活、学校生活を取り戻すためにも、希望する子供たちには打てるようにと、学校の中でも差別とかがないようにということも含めて、学校でのフォローもお願いします」(G 0815) と伝えたという。ここで彼女が重視したのは、あくまでも「メディアによるのではない、正確な情報提供」であった。

反ワクチンの先生も、(…) 正確な情報を常にお伝えできることで、もしかしたら変わっていっていただける可能性があるようだったら、ということで、そういう意味では情報提供を諦めないことは大事なのかなと思っています。(G 0815)

この語りにもあるように、インタビュイーとなったプライマリ・ケア医は、ワクチン接種プロジェクトを「スケーラブルにする」べく、正確で丁寧な情報提供をしつつ（そうすれば多くの人はワクチン接種の意義を理解するだろうという前提のもとで）、判断は情報受容者に任せるという、いわば通常の診療におけるインフォームド・コンセントを拡張した働きかけを行っていた ^17^。

### 3. 接種を準備し、実施する

次に、自身の医療施設における接種を見る。接種の準備と実施は、国や学会、行政などが出す詳細で適宜アップデートされていくガイドラインや通知を参照するという煩雑な作業を伴って進められた。

すでに何度か述べた通り、予約や登録システムの煩雑さや、待機場所の問題、後述するような経営上の問題から、接種をしない選択をする医療施設も少なくなかった。

ワクチンって 1 回接種が 2,080 円っていう今設定になっているんですけど、15 分から 30 分、様子を見なきゃいけないっていうことになっているから、そうすると、1 時間当たりにできる人数って、限られちゃうんですよね、待合室のキャパシティーの問題から考えると。そうすると、1 時間当たりに普通の患者さんが診れる売り上げよりか半減ぐらいしちゃうんですよね、下手すると。(E 0522)

接種を行う施設と行わない施設は全く無関係というわけではなく、「他に接種を行う機関があるからそこに任せて、うちではやらない」などのように、地域のなかで関わり合っていた。ある医師は、医療従事者向け接種を通じた近隣の医療施設との関係強化という興味深い事例について語る。

結構周囲の医療機関で〔医療従事者向け接種を〕やる所がほとんどなかったので、200 人ぐらい (…) やりました。(…)〔〇〇市は〕木曜日が (…) 休診のとこが割と多いんですけど、水曜日と土曜日に集中日をつくって (…) 翌日が休診 [日で、接種した医療者が] 休めるようにしたり、あと、基本的に〇〇県からの通知としては、普通〔接種を実施する〕医療機関に迷惑が掛らないように、1 つの医療機関はまとめて同じ日に受けなさいっていう通知が来ていたんですけど、そうすると〔受ける方の医療機関では多くの職員に同時に副反応が出て〕困るだろうなと思ったので、分けてもいいような感じにしたりしたので、そこはすごい感謝をされて、地域の中でのプレゼンスがちょっと上がった。(I 0621)

他方、医療機関が少ない地域では「やらない」という選択肢は事実上なく、「後がない」という使命感を持って取り組んだ。

へき地のほうの診療所は、僕らグループ診療でやっている家庭医の診療所じゃなくても、自治体立の病院が、もう俺らの所しかねえだろうって、後はないんだからっていう感覚で (…) ワクチン〔体制の整備〕もあっという間でした。(A 0621)

接種することにした医療施設では、接種に向けた組織化をする必要があった。施設によって、院内の感染対策チーム (ICT) が担当したり (D 0416)、院内ワクチンチームを設置したり (B 0416) と、それぞれの実情に応じた組織化が進められた。中にはプロジェクト化して若手医師に任せた施設 (A 0323) や、次のように担当者を募集した施設もあった。

〔ワクチン接種を〕を進めていこうっていうふうになったのが 3 月の終わりか 4 月かのあたりだったんですけど、そこで僕、いろいろ、1 年間のいろんな疲れだとか、何だとかがたまって、かなりしんどいですって言ったんですよ、朝礼とか、みんなの前で。(…) ワクチンのこととかは誰かが中心になってやってもらえると助かりますっていう、リアルな叫びみたいなのがあったんですけど、そこで (…)〔看護師の〕主任の人がやりますっていうふうにやってくれて、(…) 他に“隊長”がいるっていうだけで、かなり気が楽になって。(I 0621)

組織化し、次に取り組むのは接種のスケジューリングである。すでに述べたように、今回のワクチンは他のワクチンに比べて、扱いの難しさがあり、担当する医師や看護師、事務などの頭を悩ませた。

週に 2 回ワクチンが届くので、それを使っていく準備をスタッフとしているような状況ですね。〔1 つのバイアルを、解凍後〕6 時間以内で使い切るように、なるべく予約を 6 の倍数で取るようにはするとかですね、後キャンセル待ち希望の人はそのリストを作っておいたりだとか、自治体の方でもキャンセル待ちのシステムを作っているので、院内でどうしても見つけられない時は市の方に電話をして、キャンセル待ちを探してもらって当日に来てもらうとか、という風にして、何とか捨てる量を減らす努力はしようかなというところでやっていますが、現実は結構厳しさを感じています。(G 0508)

〔接種が〕始まると余ったワクチンをどうするか問題が (…) 結局、余ったワクチン、高齢者を回すか集団接種で今行っているところで、保健師さんが詰めに行ってくれているので、そこの場で余ったらその人に打ってしまいましょうみたいにして、どんどん回してはいるのですが、でも確かに個人のクリニックとかで、うちも個別接種するのですが、やはり余ったらどうするかとか、キャンセルが出たらどうするとか、打てない人がいたらどうするか問題が結構あって、だから、近くですぐに来られる人を何人かリストアップしておいて (…) 2 回打つというのが、スケジュールを立てる意味で〔大変で〕、では 2 回目で熱出てその日に打てなかった人の 2 回目はどこに入れるとか、かなり事務的調整コストがかかる。(F 0430)

加えて、接種において繰り返し悩みどころとして言及されたのは、在宅（往診）患者への接種であった。ここにはワクチン接種プロジェクトの「スケーラビリティ」の限界が見出せる。持ち運びについては、厚生省から「車での移動は可だが自転車は不可」などの詳細なガイドラインが示されたが、在宅患者たちの個別の条件が異なる中、「接種しに来てもらう」か「患者のところまで接種しに行く」か、あるいは両者をどう分けるか、などの選択は難しかった。もし「来てもらう」を基本方針とした場合、どうしても来られない人も出てくる。逆に接種しに行く場合は、ワクチンにロスが出てしまいうる。さらに、ワクチン接種は診療報酬が発生する「往診」ではないため、移動および接種後の観察を考えると時間のかかるワクチン接種は、収入面から見ると損失になる。加えて、訪問診療は 2 週間に 1 度というペースで行うことが多いので、間に 3 週間を空けて 2 回接種しようとすると、通常の往診スケジュールに合わず、混乱が起きてしまう、という問題もあった。

この他に、「スケーラビリティ」に関わる問題として、2 つ挙げておきたい。1 つは、高齢者福祉施設などにおいて、担当医が医師会に入っていないとか、担当医の拠点が違う自治体にあるなどの理由でワクチン配給のスピードが周囲よりも遅れてしまい、そこでクラスターが発生してしまった、という事例である。接種の遅れとクラスターとは直接の因果関係はないが、しかしいわば接種体制の「穴」のように、リスクの高い人々が取り残されていたことが発覚した出来事であり、発覚後はそのような事例が再発しないようにワクチン接種が進められた。

もう 1 つは、外国にルーツのある人々への情報提供の不十分さのことである。

外国人の方で、届いているのだけれど、どうやってアクセスしたらいいかわからない、という人も実は取り残されている、ということがわかって (…)。〔他方で〕外国人の方はコミュニティが結構しっかりしているというか、そういう人も多いので、そういうところは本当に 4〜5 人で (…)、みんな一緒に来て、一緒に打って行って、みたいな感じで、そういう人はいいのですけれど、一方でちょっと孤立している方もいらっしゃるので、そういうところは、大丈夫ですか、というような形で、特に働いていない世代の方は、市から連絡を入れてもらったりしているような状況です (G 0815)

これについて語った G 医師は、第 1 節の区分で言う①であったため、行政との連携がスムーズだったといえるかもしれない。孤立の問題は必ずしも国籍や言語に関わらず起きうるし、第 II 章でふれたワクチン忌避の問題にもつながる部分がある。そうした事態の予防と対応にも、目が向けられる必要がある。

### 4. 接種を継続するなかでの調整とバランス

プライマリ・ケア医たちは、接種を進めながら、副反応の出方や割合などを統計的・確率的に確認したり、接種の時間や対象者を調整するなど、多くの人々への接種をできるだけ円滑に進めるための工夫を行っていた。そうしたこともあり、「1 日あたり 140 人のペースでバシバシ打つ」(A 0621)、「施設とかは土曜日に行って 60 人、70 人ずつ打ったり」(I 0621)、「うちでトータル、コロナのワクチン 3,000 回打った」(I 1130) のように、接種を 1 回（一人）ずつの行為としてよりも、数十、数百、あるいは数千回（人）のような、マスで語る傾向があった。ここには、ワクチン接種がスケーラブルだという暗黙の前提を見いだせるかもしれない。

その一方で、こうしたスピード・規模で数か月にわたってワクチン接種を続けることは、医療者自身や施設に様々な影響も及ぼすことになる。特に語られたのは、通常の診療とのバランスの問題であった。次は、接種者の代わりがいない地域でのワクチン接種の事例である。

離島の診療所では午後は基本的には一般外来は休止にして、午後を要はワクチン接種に充てるというふうにしてます。ただ、もちろんワクチン接種してる間にも急患が発生した場合は、島に本当そこしかないのでやっぱり対応せざるを得なくなりますけども、基本急患だけっていうふうになる。それを多分ずっと続けることになるので、やっぱり診療体制としては少し影響が出ていると思います。あとは、ほかの病院では土日返上で医師が持ち回りで担当して、やや集団接種に近いかたちでワクチン接種を実施していくというふうにしています。やっぱり大きな影響出てると思います。(J 0521)

このように「ワクチン接種に参加し、接種率を上げること」と「かかりつけ患者を中心に、診療を行うこと」のバランスをとることはけっして簡単ではなかった。とくに第 5 波のピークで自宅待機の患者が問題となっていた 8 月の都市部の医療施設は大変厳しいところがあった。

ワクチンについても、うち、毎週 150 人ぐらい打ってるんですね。(…) 発熱外来も、たぶん〇〇区の 3 分の 1 の人〔陽性者〕を抽出するだけのことはやってると思ってるんです。(…) けれども、今度の自宅療養者〔のケア〕を本気で頑張れとなった場合は、正直どっかを削らなきゃいけないってなるんですよね。1 つは、通常診療を削るというのはもちろんあるんですけれども。この残りの 2 つを今の体制のまんま自宅療養者につぎ込むのは無理なので、実はきのう決めたんですけれども、うちはもうこれ以上ワクチンやらない。(…) ワクチンやってくれる医療機関いっぱいあると思うんで、そっちでお任せします。(E 0818)

発熱の患者さんが増えてきた中で、診療所の看護師さんとか、事務さんの中で、とにかく発熱の患者さんが来る時は、(…) やることが多い、特に事務スタッフと看護師さんのやることが多くて、医者が診察する前に、ある程度の情報を、揃えておく必要がある、ですので、電話を何回もしたりとかして、いつ来るのかとか、どうやって来るかとか、周りでどのくらいの感染の可能性が高いとかみたいなことを看護師さんがトリアージしたりですとか、事務さんは、事務さんで、保険証を、どうやって感染対策をしながら保険証を受け取るかとか、(…) ワクチンの業務が、結構、慣れては来たのですが、負担になっている中で、感染者の数が増えて来て、これは回らないという様に、一度なって、診療所の雰囲気としても、もうこのままであれば、続けていられないみたいな雰囲気になりました。(…) 所長や他の先生と相談していく中で、何を取るか、何を優先していくか、というところを検討しなければいけない段階で、やはり、このままワクチンを続けていて、発熱の患者さんを絞ったりするのか、(…) 自分たちができる範囲を少し制限しないと、自分たちは、このままですと感染するのではないか、という不安が高まってきた流れがありました。その頃が、多分、一番、診療所の中でも、(…) お昼休憩の時間が、少しどよんとする様な、そういった雰囲気がありました。(…) お昼に、また午後の人のワクチンの分を詰めるのですが、看護師さんも、また、そんな時間になっちゃった、みたいな感じでした。(C 1120)

このように、大規模なワクチン接種は、感染の波と似たようなかたちで、通常の診療（救急を含む）に対して負担を与えていた、ということができる。

### 5. 「かかりつけ」の問題化

最後に取り上げるのは、プライマリ・ケア医たちがワクチン接種のなかで見いだした、「いつものところで打ちたい」と希望する人々の存在である。こうした希望は、今回のスキームにおいては、いわばワクチン接種をノンスケーラブルにする要因だったと言える。

先週 (…) 30 人くらい〔の市民に〕レクチャーをしてきたのですが、皆さん、やはりうちで打ちたい、いつもの病院とか診療所で打ちたいという人がたくさんいて、皆、そういう風に思っていて、すごく私自身はそれが意外でした。ワクチンってどこで打っても別にあまり変わらないのではないかと私は思っていたのですが、やはり患者さんはいつもの人、慣れたところで、ワクチンを打ちたいという患者さんがすごく多くて。(C 0430)

こうした患者からのニーズに気づいたこと、さらにワクチンの供給量が限られていたり、不安定だったりすることによって、医療機関の中には、かかりつけの患者への接種を優先しようという動きも起きた ^18^。

〔線引き〕していますね、普段かかっている方に限定していますね。それで、そうではない人は、大規模なり地区がやってるところなり、に行っていただくように促すようにしていましたね。(…) 悩みますし、苦情も来るところでもありましたね。一応うちは、もう定期的にかかっている方、とか、元気な方でインフルエンザだけ来る方とかもいらっしゃるのですよ。そういう方は、65 歳以上の時に関しては、2 年連続で来ている方とかにしていました。(…) 年齢層が下がって〔く〕ると、元気で受診していない方もおられて、たまに風邪をひいたら、数年に 1 回来る、というような人も心理的にはうちをかかりつけと思っている方もいるのですよね。最初、それを65歳以上のルールに適用して、電話で対応していたら、やはり 1 人めちゃくちゃ怒った人がいて、少しルールを変えなければいけないね、と言ったまま〔まだ〕変えていないです。(H 0815)

そうした動きの中で明らかになったのは「かかりつけ」とは誰なのか、という定義の曖昧さであった。

やっぱりここで、かかりつけ医っていう言葉が (…)、ただ単にその患者さんが、かかりつけっていうのを、片思いで思っているだけかもしれないし、そこは本当にかなり曖昧なわけですよね。かかりつけだとした場合に、責任を持ってワクチンを必ず打ってあげられるのかっていうと、あげられないわけですよね。(…) 逆に言うと、例えば本当にかかりつけ登録制っていうふうにしていて、あなたのクリニックはここのうちの健康管理をちゃんと担うんですよっていうふうに言われているような、お墨付きがあるんであれば、そこに登録している患者さんは、まず、うちで必ずワクチンをやるんで言ってくださいねみたいなことが言えますよね。(…) でも、フリーアクセスなんですよ、日本は。(…) 僕らはかかりつけだと思って、この人の分のワクチン用意しておいたけど、「あなた、かかりつけじゃないわよ、他でやっちゃったわよ」なんて言われちゃうと、僕〔は〕「どうするの、このワクチン?」、てな話になっちゃうわけですよね。なので、この曖昧さっていうのがかなり、ワクチン一つ取ってみても、すごく今回のオペレーションを、うまくいってないというか、みんなの不満が大きくなるような一つの原因になっているのかなと思います。(E 0522)

以下の語りは、こうした課題を的確にまとめている。

コロナ禍はもっと複雑ないろんな問題、課題をですね、プライマリ・ケアにも提示している気はします。主治医不在であるとか、ワクチンをどう進めていくかとか、(…) 若年者になればなるほど、かかりつけ医なんて持っていない人がほとんどなので、だからこそ日本のプライマリ・ケア・システムのより強化とか、そういうのは〔必要だと〕僕は思いますし、海外のような登録制というか、ペイシェントリスト・システムっていうのが、やっぱり日本でも必要なんじゃないかって、改めて思いますしね。自分たちの主治医、家庭医はあの先生っていうのが決まっていれば、その先生に本当にワクチンについて聞くとか、どう行動すればいいとか、そういう助言を得るとか、そういうようなことがより容易になれば、もっと不適切なことだったり、あとはワクチンに関しての変なデマ情報ですね、ああいうようなことも防げるんじゃないかなというふうに思います。　だから、(…) より一層このコロナ禍の中で、日本ではプライマリ・ケア・システムの構築だったり、強化が必要かなと思いました。(J 0903)

おそらく今回はより早く、より多くの人に接種するために、医療機関や大規模接種会場などを同時にオープンし、「どこでも打てる」ように自由度を高める方針が取られた。だが実際には、とりわけ高齢者の中に「いつもの場所」で接種したいという希望が見られた。さらに、ワクチン供給が不安定で、かつ人々の接種ニーズが（一時的にであれ）接種のキャパシティーを超える中で、接種現場においてはいわば「トリアージ」が必要といえるような状況が生じていた。それによって、プライマリ・ケア医の視点からは、ワクチン接種のスキームではあまり想定されていなかった、いわば守るべき対象の明確化として「かかりつけ」という範囲がより具体的で喫緊のものとして浮かび上がっていたのである (cf. 木村ほか, 2022)。

## V. 考察と結論――プライマリ・ケア医にとってのワクチン接種

### 1. 接種を振り返って

以上、新型コロナウイルス感染症におけるワクチン接種について、プライマリ・ケア医のインタビューから明らかになったことを整理した。プライマリ・ケア医たちは、計画の見通しが悪く、市民も意向が定まらない中、それでも接種率を上げることがよいことであるという（職業的）信念のもと、同僚や患者、社会に向けてワクチンの意義や接種の手続きなどを説明しつつ、現場でも様々な調整をしながら、経営的利益ではなく、社会（地域やかかりつけ）への大事なサービスとして、それぞれのキャパシティーの許す限りで接種を行っていた。

その過程では、行政からの情報が直前まで分からないことにストレスを感じたり（第 IV 章 1 節）、反ワクチンの言動を見て落ち込んだり（同 2 節）、感染拡大期における必要な業務の多さで疲弊したり（同 4 節）というような苦しみも経験していた。

しかし、2021 年 11 月時点で振り返ってみたとき、インタビュイーであるプライマリ・ケア医たちは、今回の接種プロジェクトが基本的に成功であったと考えている。その要因の 1 つには、接種率が挙げられていた。

何だかんだ、でも、公衆衛生的には、ワクチンに関しては成功した部類に入るかなと思うのですが。オリンピックがあったからなのかも知れないですが。(D 1118)

よく、2・6・2 の法則と言いますが、2・6 のところまでは、全て打っているので、残りの 2 は、もう仕方がないと思うと、2・6 で 8 割、ほぼ行きますので、すごく打ったねと言うのが、正直なところです。(B 1118)

もう 1 つは、インフルエンザ等、他のワクチン接種への好影響である。プライマリ・ケア医たちは「コロナのこともあってか、ワクチンに対して、比較的、やはりポジティブな思いを持っている人たちも、それなりに出てきている」(B 1118) という実感をもっていた。

本当に「ワクチン、何も打ったことがない」という患者さんがコロナで初めて打ったので、何か、予防注射みたいなものは、打っても良いものかという雰囲気になって、今まで、頑として打たなかった人が、今年は「では打つか」みたいな雰囲気になっている人は、何人かいます。(C 1120)

とはいえ、約半年間という急なスピードで進められた接種プロジェクトに対し、懸念の声も聞かれた。

ワクチンに関しては、パターナリスティックに進んだなという印象があって (…) でも、私たちは、普段の医療の中で、特に私とか大事にしたいのは、あまりパターナリスティックに (…) 押し付けるというよりは、やはり、そこできちんと意見交換しながら、分からないところは、分からないというところの中で、そこをペンディングする人がいた時に、それは、それとして尊重して進んで行くみたいなところが、結構、大事なのですが、割とその議論が、今回、無理やり進んだところもなくはないだろうなと思っていて、そこが今後のその対話をしていく医療に、与える影響がいろいろありそうだなと思って、少し懸念しています。(B 1118)

この懸念はインタビュイーのうちの 1 人から発されたのみなので一般化には注意が必要である。とはいえそこには、プライマリ・ケア医たちが普段から持っている基本的な姿勢や価値観と今回のワクチン接種の進め方が必ずしも一致していたわけではないことが示唆されている。新型コロナウイルス感染症に対しては、ワクチン接種以外でも、画一的・大規模な検査や、患者との接触を減らしながらの診療が行われており、個別的で丁寧な対応を旨とするプライマリ・ケアのあり方との間に葛藤を生じている。本稿で見てきたプライマリ・ケア医たちの実践は、たんにワクチン接種を「スケーラブルにする」ことだけを目指したのではなく、そうした価値観との葛藤にいかに折り合いを付けるか、ということも含まれていたと言える。「かかりつけ」の問題化も、そうした中で捉えることができるだろう。

### 2. 知見として――ワクチン接種を「スケーラブルにする」ために

最後に、今回のインタビューから得られた知見をまとめておきたい。

大規模なワクチン接種は、本稿で用いた言葉を使えばスケーラブルに行われることを前提としたプロジェクトである。今回の新型コロナウイルス感染症に対するワクチン接種プロジェクトは、2009 年の新型インフルエンザなど、過去の感染症流行の経験をもとに計画され、実施されはじめたものであった。だが、実際には様々な部分で問題が発生しており、そのうちいくつかは、このプロジェクトが実質的にはノンスケーラブルな部分を含んでいたことを示していた。そのため、様々な現場において、このプロジェクトを「スケーラブルにする」ための試行錯誤が行われた。

プロジェクト遂行上の問題としては、まずは計画や方針が現場まで浸透しておらず、また変更も多く、さらに根幹となるワクチン供給も不安定であったため、現場に大きなストレスを与えたことが挙げられる。政府は災害対応と同様に、都道府県や自治体をワクチン接種計画の実施主体としたが、その結果スピードや効率性に大きなばらつきが出てしまったことは否めない。本調査から見えたのは、現場の医師の間でも、これまでの実績にもとづく自治体や医師会との距離によって、情報量が異なり、見通しの立てやすさに差が出ていたということである。行政や医師会と医療機関の間のコミュニケーションがより円滑になり、また情報共有が進むような工夫が必要である。

加えて、今回のインタビューでは、ワクチン接種におけるノンスケーラブルな事象として、在宅患者への接種、接種の「穴」や外国人などの問題も指摘された。全体としての接種率の向上だけでなく、感染や重症化リスクのことを考えるなら、こうした事象に対してきめ細やかな対応が必要である。在宅患者への接種に関しては、より詳細・具体的にグッド・プラクティスの収集が進められるべきであろう。「穴」に関しては、医療機関や行政のみが対応するのではなく、地域内のより多様なアクターの連携によって早期に発見し対応できることが望ましいと考える。

また本稿からは、プライマリ・ケア医たちは、たとえ自身のワクチンへの希望が揺らいでいたとしても、ワクチン接種の意義を認め、ワクチンの効果や予約等に関わる情報提供を続けていたことが指摘できる。これは実際の接種と並んで、スケーラブルであることを前提としながら、実際には決してそうではない様々な事態を含んだプロジェクトをなんとか調整し、円滑に進めるための重要な仕事であり、かれらがこうした仕事をしていたことをきちんと評価することと同時に、現場にしわ寄せがいかないような体制づくりが必要である。

このことの重要性は、ワクチン業務が、医療施設の組織（医師以外も含む）や日々の業務、経営に対して負の影響を与えていたことからも指摘できる。とりわけ感染者数が増加し、かれらへの医療行為が必要となった状況では、医療現場にかかる負担はきわめて大きなものであった。この負担のため、医療資源の豊富な地域ではワクチン接種を行わないという選択肢をとる医療機関もあったが、医療資源の少ない地域ではその選択肢は事実上存在しなかった。

こうした事態は、プライマリ・ケア医たちの「スケーラブルにする」努力にも限界がありうることを示唆している。このことと、「基本的には接種を勧めるが、接種を拒む人は個別の判断だとして許容する」というプラクティカルな態度はつながっていると考えられる。「パターナリスティック」への危惧もあったように、プライマリ・ケア医たちは基本的な姿勢として個別的で丁寧な対応を心がけている。その姿勢と、多くの人々へのワクチン接種というプロジェクトをよりよいかたちで折り合いを付けるにはどうすればよいか、より深く検討することが必要である。

以上が今回のインタビューに基づく知見である。ただし、今回は、行政、保健所、他職種などの業務や相互的な関わりや、ワクチンに対して躊躇ないし拒否した医師たちについては論じることができなかった。それらについての調査や考察も行われるべきである。

感染症対応は計画とテストを繰り返しつつ改良されていく( Caduff, 2015)。本稿で明らかになった実情やそこから示された知見が、今後の計画の改善につながることを期待する。

## Data Availability

本論文の研究結果の基礎となるデータであるインタビューの回答は、本論文に含まれている一部を除いて、公開することができない。インタビューの内容が対象者の私的な情報や対象者を特定できる情報、医療実践上の問題など対象者に不利益を生じうる情報を多く含むために、データを非公開とすることが倫理審査に基づく同意書に書かれていることが、その理由である。同分野の研究者や査読者がデータの閲覧を希望する場合には、データの利用目的と方法を明記の上、著者 ( kimura.shuhei.ge@u.tsukuba.ac.jp) に連絡されたい。

## Footnotes

^1^
その傍証として、ワクチン接種を行うことが医師以外の複数の専門職に認められている（どこまでかは国ごとの法制度が規定する）ことを挙げることができる。

^2^
配置という語はフーコーによる装置 (dispositif ) や環境 (milieu) という概念をもとにしている（ 浜田, 2017: 634-5）。

^3^
以下、インタビューを引用する際は、(　) 内に表 1 に示された略号とインタビュー日を 4 桁の数字 (MMDD) で記載する。語りをそのまま掲載する場合は「　」で括る。(…) は省略、〔　〕は調査者による補足や注釈を示す。

^4^
厚生労働省「新型コロナウイルスワクチンの供給に係るアストラゼネカ株式会社との契約締結について」 https://www.mhlw.go.jp/stf/newpage_15422.html　 2022 年 10 月 6 日閲覧。

^5^
手引きはその後、本稿執筆時点まで繰り返し改訂されている（投稿時点での最新版は 2022 年 8 月 1 日に出された 8.3 版である）。 厚生労働省「新型コロナウイルス感染症に係る 予防接種の実施に関する手引き」 https://www.mhlw.go.jp/content/000971375.pdf　 2022 年 9 月 16 日閲覧。

^6^
厚生労働省「新型コロナウイルス感染症に係る予防接種の実施に関する手引きについて」 https://www.mhlw.go.jp/stf/seisakunitsuite/bunya/vaccine_notifications.html　 2022 年 10 月 6 日閲覧。

^7^
厚生労働省「医薬品医療機器等法に基づく新型コロナウイルスワクチンの特例承認について」 https://www.mhlw.go.jp/stf/newpage_16734.html　 2022 年 10 月 6 日閲覧。

^8^
厚生労働省「新型コロナウイルスワクチンの 接種体制確保について 自治体説明会③」 https://www.mhlw.go.jp/content/10906000/000740274.pdf　 2022 年 10 月 6 日閲覧。

^9^

ただし、ワクチンの卸業者等との契約は都道府県が行うこととなっている（厚生労働省「新型コロナウイルス感染症に係る予防接種の実施に関する手引きについて」 2020 年 12 月 17 日 https://www.mhlw.go.jp/content/000714250.pdf）。 2022 年 9 月 16 日閲覧。

^10^

「首相、高齢者ワクチン接種「7 月末までに」 」 『日本経済新聞』 2021 年 4 月 23 日付, https://www.nikkei.com/article/DGXZQOUA23CMF0T20C21A4000000/　 2022 年 10 月 6 日閲覧。

^11^

「菅首相会見 ワクチン接種 1 日 100 万回を目標とする考え」 『NHK』 https://www3.nhk.or.jp/news/html/20210507/k10013017981000.html　 2022 年 10 月 6 日閲覧。

^12^
内閣府の実績データ ( https://www.kantei.go.jp/jp/headline/kansensho/vaccine.html) に基づく。

^13^
診療所を中心に接種を進める「練馬区モデル」がよく知られる( https://www.mhlw.go.jp/content/000738265.pdf)。また、今回のインタビュイーの勤務地の自治体でも、様々な取り組みがあった。この自治体では、接種希望者の予測をするためにアンケートを行い、見通しを立てている。とくに子供への接種について不安の声も上がる中、まず受験生から接種することで批判を回避して接種を進めた (G 0815)。さらに、子供の接種に関して「接種する場所をパーテーションで区切って個室にしたりとか、昨日はパーテーションの内側にすごく景気のいいお祭りの写真とか、大画面で 4 方向に張り出したりして、(…) 緊張感を感じないように打つようにするとか、(…) 受験生が健康に過ごすためにこうしましょう、みたいなリーフレットを配って、(…) スマホを見ないようにしてもらう〔接種直後にスマートフォンを凝視していると気分が悪くなりやすいため〕とか、後は医療者向けのコメントをポストイットに書いてもらっていて、そこに貼っていってもらうとか、そういう細かい企画を」行った。これは「受験生と一緒に保護者 1 名も接種できます (…) という感じで (…) お得感があったので、ちょっとインセンティブが高まった」 (G 0815) のではないかという。 さらに、「病院に行けない赤ちゃんを育てている家族の人に、『楽々接種』とか言って、医師がオンラインで問診をして、保健師さんが家庭を訪問して、接種をしつつ子供の状態を見るみたいなサービス」(G 1130) も行っていた。

^14^
ただし、子どものワクチン接種、とくにインタビュイー自身の子どもの接種については、躊躇する語りもあった。

^15^
他のワクチンで使った予約システムを流用しようとしてうまくいかなかった、という事例も聞かれた。予約システムは、医療機関が日常使っているものを使ったり、企業のサービスを利用したりする場合もあった。また、トラブル多発のために途中から自治体のシステムを導入するという事例もあった。

^16^
ある医師は「やはり医療者は身に染みているのです、この大変さを、なので、結構、打たないという人は、私の周りにはいないです、今のところ」(F 0430) と、接種しないという選択肢が事実上なかったことを語っている。

^17^
ただし、統計的なレベルでの接種数は、マスメディアによる報道や感染の「波」などの影響を受け、かれら個々人の関与できる範囲を超えて増減していたということもまた事実である。

^18^
看護師たちにその傾向が顕著だったと指摘する語りもある (C 1120)。
