# Supplementary material for: How have Japanese primary care physicians carried out vaccinations against COVID-19? : Attempts at making the non-scalable ‘scalable’
Source: F1000Res. 2023 Feb 24;11:1268. [Article in Japanese] Originally published 2022 Nov 8. [Version 2] doi: 10.12688/f1000research.126366.2 (PMC10448142; doi:10.12688/f1000research.126366.2)
Supplement: Peer review history

# Peer review: Reviewer response for version 2

**DOI:** 10.5256/f1000research.144189.r177949

**Published:** 2023-08-23

**Authors:** Miho Ushiyama

**Affiliations:** 1 Faculty of Human Relations, Otsuma Women's University, Tokyo, Japan

　本論文は、新型コロナウィルスのワクチン接種について、これにかかわった全国各地のプライマリ・ケア医を対象にオンラインの聞き取り調査を行い、そのデータを文化人類学的視点から分析したものである。筆者も述べているように、文化人類学の研究には、ワクチンを扱ったものもそれなりの数があるが、その多くが患者のワクチン受容の問題に焦点を当てており、医師の側からワクチン接種の実践について調査した研究は、管見の限りでは見当たらない。そうした意味で、医師の側からみたワクチン接種の実践についての調査を行い記録を残しておくことには、一定の意義があると考えられる。

 　しかし、下記に述べるいくつかの点から、本論文には改善の余地があると考えられるため、このような判定とした。査読者の専門が文化人類学／医療人類学であるため、ここでは、文化人類学的な観点から指摘を行いたい。

 　本論文では、何をもってこれを文化人類学的な調査とするのか、という点が不明確な点が問題ではないかと考える。パンデミックのため文化人類学の本懐であるフィールドワークができなかったことが一番の理由だが、それでは何をもって本調査を文化人類学的な調査と呼ぶのか、その根拠がやや乏しいと感じる。本論文では、「上記のような姿勢でインタビューをすることでできるだけ対象者の視点に接近したこと、また既存の集合的カテゴリーや枠組みで対象者を理解するのではなく、対象者の言動のなかから現れてきた差異と共通性に即して集合的カテゴリーのあり方を、文化人類学の先行研究を手がかりにして考え直そうとする点で、文化人類学的な研究だと言える」と説明されているが、インタビューの姿勢、文化人類学の先行研究を手掛かりにしているという点のみで文化人類学の調査としてよいのだろうか。

 　以下、３点、本論文を文化人類学的な調査として成立させるための提案をしたい。

 １．論文全体を貫く視点・調査者たちの立場について

 本論文は、（文化人類学者と医師が共同で行った調査をもとにしていることも関係しているのではないかと推測されるが）論文全体を貫く視点が、文化人類学的というよりは、医師の視点を強く反映しているように感じられる。とくに、「Ⅴ. 考察と結論」の「2. 知見として―ワクチン接種を「スケーラブルにする」ために」の節は、いかに今後ワクチン接種をスケーラブルなものにすることができるか、医師にとって有益だと思われる知見をまとめた形になっている。こうしたまとめ方を読むと、本調査が文化人類学的な調査であるとの意義がみえにくくなってしまうと感じられる。

 科学や医療分野における文化人類学的研究の意義のひとつに、こうした分野の知見を異化したり相対化したりするというものがあるが（Epstein 1996, ラトゥール1999, モル 2020）、こうした視点のもち方も、調査を文化人類学ならしめる要素のひとつであると考えられる。本調査では、そうした文化人類学的な視点から、医師の実践を相対化するという視点はとられておらず、医師とほぼ同一の視点から全体を通して分析がなされている。もしもこうした視点が、調査の構造上やむを得ないものであったり、むしろ意図的にそのような書き方をしたりしているのであれば、この論文がどのような立場・視点から書かれているものなのかを明確に説明し、それでもなぜこの調査が文化人類学的なものと呼びうるのか説明がほしい。

 ２．「スケーラビリティ」概念について

 　本調査が文化人類学的な調査とされる理由のひとつに、文化人類学的な概念であるアナ・チンの「スケーラビリティ」の概念が分析の軸となっていることが挙げられるだろう（チン 2019）。しかし、本調査の内容は、「スケーラビリティ」概念を持ち出さずとも説明できる内容であり、本調査を文化人類学の調査として成立させるという目的のためにこの概念が用いられている印象がある。先述の内容とも重なるが、本論文がほぼ医師の視点と同一の視点から書かれているため、文化人類学的概念を使って、医師の視点とは異なる視点から分析するということ、そのためにはどうしてもこの文化人類学的な概念が必要だ、というような、必要性のある文化人類学的概念がうまく探し出せていない印象がある。もしも、「スケーラビリティ」概念ではなく、ほかの概念を軸に、医師とは異なる視点から分析ができるのであれば、それが望ましいのではないか。

 ３．分析の観点：ノンスケーラブルな部分について

 　しかし、もし「スケーラビリティ」概念を使いながら、より本論文を文化人類学的なものにしていくのであれば、以下のような提案をしたい。

 「Ⅴ. 考察と結論」の「2. 知見として―ワクチン接種を「スケーラブルにする」ために」の節は、将来に向けてスケーラブルな計画を改善していくための知見を集めているという形にはなっているが、よく読めば、ワクチン接種におけるノンスケーラブルな部分を課題として抽出した内容になっている。こうした個別的で偶発的なさまざまなノンスケーラブル的問題を丁寧に取り扱うということは、文化人類学的な視点の特徴であるともいえる。節のタイトルや内容のまとめ方について、「一見スケーラブルに動いていたようにみえるワクチン接種の裏のノンスケーラブルな部分に目を向けることで何が見えてくるのか」、といった書き方を意識すると、より文化人類学的な視点が入ってくるように思える。

 また、「１．接種を振り返って」で書かれていた、ある意味ノンスケーラブルな姿勢をもつプライマリ・ケア医が、今回のワクチン接種のようなスケーラブルな実践にかかわるときに葛藤を抱えるという点は、興味深かった。こうした細やかなノンスケーラブルな部分を拾い上げるという視点に、文化人類学的と呼びうる点があるように感じられるので、こうした箇所をもう少し強調して、分析の視点に文化人類学的な要素を入れるとよいのではないか。

 Epstein, S: Impure Science――Aids, Activism, and the politics of knowledge. Berkeley: University of California Press.1996.

 チン, A: マツタケ――不確定な時代を生きる術. 赤嶺淳訳. みすず書房. 2019.

 ラトゥール, B:科学が作られているとき――人類学的考察. 川崎勝・高田紀代志訳. 産業図書.1999.

 モル, A: ケアのロジック――選択は患者のためになるか. 田口陽子・浜田明範訳. 水声社. 2020

（該当する場合は要回答）統計分析および解釈は適切ですか。

対象外（統計を使っていない）

本研究は明確かつ正確に提示されたものであり、最新の文献を引用していますか。

はい

方法と分析について第三者による再現が可能となるよう十分な詳細が提示されていますか。

一部該当

結果の基礎となるソースデータはすべて入手可能で再現性を十全に保証していますか。

ソースデータは不要

結論は結果により妥当な裏付けを得ていますか。

一部該当

研究設計は適切で学術的価値がありますか。

一部該当

Reviewer Expertise:

文化人類学／医療人類学

I confirm that I have read this submission and believe that I have an appropriate level of expertise to confirm that it is of an acceptable scientific standard, however I have significant reservations, as outlined above.

# Peer review: Reviewer response for version 2

**DOI:** 10.5256/f1000research.144189.r169061

**Published:** 2023-07-11

**Authors:** Tatsuya Mima

**Affiliations:** 1 Ritsumeikan University, Kyoto, Japan

本研究は、日本におけるプライマリ・ケア医の行ったワクチン接種の意義について、プライマリ・ケア医に対するインタビュー調査をまとめたものである。記録という面では一定の意義はあるが、研究目的やその作業仮説は明確に記載されているとはいえない。研究目的のさらなる明確化が必須である。

 １．日本におけるワクチン接種の実践を考察する上で、プライマリ・ケア医を対象として選定した基準が明確ではない。職域接種などの場合についてどう評価するか、またプライマリ・ケア医がワクチン接種のどの程度の割合を担ったか（インタビュー当時までのデータでよい）を記載したうえで、研究対象者選定の根拠を明記する必要がある。

 ２．「しかし、将来的なパンデミックの際に起きうる問題に備えるうえで、現場での対応に関わる記録を残すことには意義があるはずだと、私たちは考える」

 序文で、このように述べられているが、たんなる「考え」に過ぎず、その根拠が示されていない。

 ３．「接種の増加による集団免疫がスムーズに実現できるわけではないこと」

 　上記以外でも、集団免疫があたかもワクチン接種の目的であるかのような誤った記載が散見される。また、人類学的な研究という点からは、医療者（インフォーマント）の信念としての「集団免疫」に対しては、COVID-19で実現可能かどうかについて医学界内部でも議論があることを踏まえて、研究者は相対化した観点に立つべきとも考えられる。

 ４．スケーラビリティの概念で分析しようとする点では野心的だが、チンの用法と一致しているかどうかには疑問が残る。むしろ、通常のシステム・エンジニアリングやITでいう「拡張性の高さ」に近い概念になっているのではないか？

 分析概念であるので、スケーラビリティについては本論文での用法を厳密に定義したうえで、論文全体の構成を再考する必要がある。

 ５．上記に関連して、そもそも、ミクロレベルでの「ワクチン接種の実践」そのものへの著者らの見方が、「ワクチン接種の率を上げるべき」という医療者の信念に引きずられている印象を受ける。たとえば、プライマリ・ケア医の信念からは距離を取り、「希望者にワクチンを接種し、非希望者にワクチンを接種しない」というのが現実で行われていた実践であるという立場に立てば、考察での「ワクチン接種をスケーラブルにするために」という節は内容的に不適である。スケーラブル・ノンスケーラブルは記述概念であって、規範ではないはずである。

 ６．ワクチン接種のスキームとして、「「接種する者」と「接種される者」「接種するワクチン」および「接種場所」の 4 つの要素」を挙げているが、これは医療を医者―患者関係の枠組みでのみ理解する古典的な生物医学の狭い枠組みにとらわれすぎているのではないか？　

 Biomedicalization（Clarke）の枠組みを例にとれば、少なくとも、テクノロジー的側面としてはmRNAワクチンのロジスティクス（輸入の確保、運送体制の整備、配分の政治問題化）、ICTとの関連としてSNSやマスメディアの影響があることを述べたうえで、本研究では、ミクロレベルでの4つの要素に焦点を絞るというステップが必要と思われる。なお、これは、スケーラビリティでのミクロレベルとマクロレベルをこの論文の中でどう定義しているかが明確でないこととも関連している。

 ７．ワクチン接種無料化の影響、接種当たりの実費用など、経済システム上でのワクチン接種の意義について、簡単に述べることが望ましい。プライマリ・ケア医にとって、経済的動機がまったく存在しないと想定するのは非現実的である。

 ８．「かかりつけ医」については、素人の考える「かかりつけ」概念と保険診療上での地域包括や機能強化加算と関連した「かかりつけ」概念を整理して記述することが、読者に対して親切である。

（該当する場合は要回答）統計分析および解釈は適切ですか。

対象外（統計を使っていない）

本研究は明確かつ正確に提示されたものであり、最新の文献を引用していますか。

はい

方法と分析について第三者による再現が可能となるよう十分な詳細が提示されていますか。

はい

結果の基礎となるソースデータはすべて入手可能で再現性を十全に保証していますか。

はい

結論は結果により妥当な裏付けを得ていますか。

一部該当

研究設計は適切で学術的価値がありますか。

一部該当

Reviewer Expertise:

医療社会学

I confirm that I have read this submission and believe that I have an appropriate level of expertise to confirm that it is of an acceptable scientific standard, however I have significant reservations, as outlined above.

# Peer review: Reviewer response for version 2

**DOI:** 10.5256/f1000research.144189.r164430

**Published:** 2023-04-17

**Authors:** Maho Isono

**Affiliations:** 1 Tokyo University of Foreign Studies, Tokyo, Japan

査読に対するご回答を御礼申し上げます。

 1回目の査読に対して行なっていただいた解説及び加筆修正をもって、採択の判断とさせていただきます。

 本論文の意義は継続的に複数のプライマリケア医へのインタビューを行ったこと、及び医師との共同研究を実施した点にあると考えるため、更なる加筆修正を求めることは致しませんが、次の点に関しては引き続きご一考いただきたいと思います。

 「既存の集合的カテゴリーや枠組みで対象者を理解するのではなく、対象者の言動のなかから現れてきた差異と共通性に即して集合的カテゴリーのあり方を、文化人類学の先行研究を手がかりにして考え直そうとする点で、文化人類学的な研究だと言える」という方法の箇所に記載された点についてです。

 こちらですが、（１）社会学や看護学などの質的研究でも同様の視座が取られていること、（２）参照した先行研究が哲学や社会学なら、それらは哲学的研究、社会学的研究になってしまうのか、という問いも含め、文化人類学の研究を学問の外に開いていく際の難しさと課題を感じました。

 「以上のやり方で、文化人類学の研究論文としてまだ問題があるようでしたらご指摘いただければ幸いです。」といった形で押し切ってしまうことも一つの手かと思うのですが、「文化人類学的な研究」とは何かを文化人類学に関心を持つ方々に本論文を通じ紹介するのであれば、類似した学問、研究法との差異をもう少し丁寧に説明してもよかったのではと思います。

（該当する場合は要回答）統計分析および解釈は適切ですか。

対象外（統計を使っていない）

本研究は明確かつ正確に提示されたものであり、最新の文献を引用していますか。

一部該当

方法と分析について第三者による再現が可能となるよう十分な詳細が提示されていますか。

いいえ

結果の基礎となるソースデータはすべて入手可能で再現性を十全に保証していますか。

一部該当

結論は結果により妥当な裏付けを得ていますか。

一部該当

研究設計は適切で学術的価値がありますか。

一部該当

Reviewer Expertise:

文化人類学、医療人類学

I confirm that I have read this submission and believe that I have an appropriate level of expertise to confirm that it is of an acceptable scientific standard.

# Peer review: Reviewer response for version 1

**DOI:** 10.5256/f1000research.138769.r155206

**Published:** 2023-01-11

**Authors:** Maho Isono

**Affiliations:** 1 Tokyo University of Foreign Studies, Tokyo, Japan

**【総括】**

 本論文はコロナ禍の初期より国内のプライマリ・ケア医に対して継続的に実施されてきたインタビューデータに基づいており、まずその点において著者が集めた語りは大変貴重であると考えます。また本研究が、文化人類学者と医師の協働であるという点も価値が高いでしょう。

 したがって本論文が意義と主張する「出来事を記憶に留める」という点においても、このように集められた語りにしかできない提示の仕方があるはずであり、その点においても意義を感じます。

 しかし１つの論文として成立させるという視点において、考え直すべき重要な点がいくつかあると査読者として評価致しました。その点に関して、下記列挙いたします。

 なお本査読は、枠組みに関する大きな見直しを提案するものであるため、事実関係や表現の仕方といった細かい部分に関しては一旦差し置いたことを予め書き添えます。

**【Major revision I】「人類学の方法」とは何か**

 【2.方法】の箇所に「人類学の方法に基づく」「文化人類学の手法に基づいているため、厳密なコーディングを行っているわけではない」と記載されています。それではこの方法は一体いかなるものなのでしょうか。

 このことが不明瞭なまま書き進められていることは、（１）本稿がF1000Researchに投稿されたこと、（２）【２.方法】がCOREQを参照しながら書かれていること、（３）質的調査一般における本稿の位置付けの３点において問題であると思われます。以下その問題点をそれぞれ具体的に記します。

**（１）本稿がF1000Researchに投稿されたこと**

1. F1000Researchの趣旨および投稿されている論文を踏まえると、本論文は文化人類学者(*)以外の読者に向けて執筆されたものと思われます。それを踏まえると、「人類学の手法に基づく」、「文化人類学の手法に基づく」というだけでは説明が不十分ではないでしょうか。文化人類学について門外漢の読者にもわかるような方法の説明を加筆すべきであると思われます。またその際には、「文化人類学の手法に基づくと厳密なコーディングが不要になる」理由も加筆ください。それがないと、GTAなどを用い厳密なコーディングを実施する研究者をも混乱させてしまうと思われます。  
2. 一般に「人類学」というと自然人類学や考古学も含まれます。したがって本論文における用語の不統一は読者を迷わせるのではないでしょうか。用語を統一し、それが何を指すかを明瞭にすべきと考えます。（*本査読では、「文化人類学」に「社会人類学」も含ませています。）

**（２）COREQを参照したことについて**

 COREQは、チェック項目を全て満たすべきガイドラインではありませんが、大枠において記載すべきものは掲載されていると私は理解しています。その記載すべき項目の一つが理論的枠組みであり、その例として、GTA、エスノグラフィー、現象学、内容分析などが提示されています。COREQに従うのであれば、「人類学の手法に基づく」理論的枠組みが如何なるものなのかの解説は必須なのではないでしょうか。

**（３）質的調査一般における本稿の位置付け**

 本稿は、看護研究などで広く行われる質的記述的研究、あるいは質的帰納的研究（半構造的なインタビューガイドを用い、同一インフォーマントに複数回のインタビューを行い、コーディングをし、カテゴリーを作る）と相当に類似しています。例えば【方法】には下記の記載がありますが、文化人類学の手法に基づくという箇所以外は、看護研究で行われる質的研究の方法とほぼ同じです。

*録画・録音したインタビューを文字起こしし、そこからワクチンに関わる部分を抽出し、内容のまとまりごとに分類した（文化人類学の手法に基づいているため、厳密なコーディングを行っているわけではない）。その整理したものをもとに、時系列に沿って大きな動きを把握するとともに、傾向やパターン、および個々の特異性を分析するという作業を行った。*

 また方法を読むと、質問票を用いない非構造的なインタビューを行ったとのこと。これを踏まえると、看護学で実施されているような質的研究に達する前の準備段階、あるいは本格的なエスノグラフィーを行う以前の報告書といった位置付けが最も適切なように思えます。

 この辺りが査読者の思い違いであるならば、その点がわかるよう加筆ください。

**（４）オンラインのみのインタビューがどのように(文化？）人類学足り得るのか**

 査読者が文化人類学者であるゆえの問いですが、私個人は、文化人類学の醍醐味は、ある地域や特定の個人に特化した深さと詳細さ、およびそこから引き出される包括的な人間/世界理解（調査地が複数の場合は、それらの繋がりをあらわにする）であると考えています。

 しかし本研究は、全国に散らばるプライマリ・ケア医へのオンラインインタビューであるため、地域性に基づく分析はなしえず、かといって日本という地域に特化した分析が行われているわけでもありません。またプライマリ・ケア医に特化した研究とも言い難いとも考えます(Major revision III参照）。

 状況的にオンラインでしか調査が行えなかったことは重々承知の上ですが、どのような理由で本調査が人類学的なのかをもう少し記載してください。

**【Major revision II】外的妥当性と内的妥当性のいずれもが担保されていない**

 質的調査におけるデータの妥当性は、文化人類学の外側では常に議論される問題です。特に、本稿が文化人類学の外側の読者に向けられていると仮定すると、本論文におけるデータの外的妥当性・内的妥当性が共に担保されていないことはクリティカルな問題ではないでしょうか。

 質的調査の場合、外的妥当性の提示は統計調査に比すると説得力に欠けますが、内的妥当性の担保はある程度可能であると査読者は考えています。

 しかし本稿は、非構造的なインタビューにより得られたデータをもとに分析がなされているため、結果で提示されている意見が、個人の意見なのか、インフォーマント全体に共有された意見なのか、プライマリ・ケア医に特徴的な意見なのか、あるいは調査者が重要と思った個人の意見なのか、といった判断もつけることが難しい状態です。このため読者はここに紹介されている語りをどの観点から読めば良いのかわからなくなってしまうのではないでしょうか。

 複数人の意見の場合は（　）書きで該当者が記されておりますが、これらは同一質問紙を用いた調査ではないため、調査者の会話の流れや、質問の仕方がたまたま似たような回答を導き出した可能性も想像されるでしょう。このような点を踏まえると、提示されたデータは内的妥当性という観点からも説得力に乏しいのではないでしょうか。

 データの価値を担保するためにも、これらデータをどのような観点から、どのように読み解けば良いのかのガイドを入れて欲しいです。

 *ここでいう外的妥当性、内的妥当性については(Malterud,2001) *を参照のこと。*

**【Major revision III】提示されたデータが「プライマリ・ケア医」の視点であることをどのように担保するのか/ 「プライマリ・ケア医」の視点とは何なのか**

  

1. 本邦で紹介されたプライマリ・ケア認定医へのインタビュー結果を、プライマリ・ケア認定医の視点や実践と呼ぶことについては留保が必要ではないでしょうか。例えば、本稿で言及がある、ワクチンへの疑念を示す人々に対しどのような説明をするか、所属施設においてワクチンを接種するスキームをどのように作るかは、プライマリ・ケア医に限られたものではなく、接種に関わった医療職全てが抱えた悩みと考えます。だとするならば、ここでいう「プライマリ・ケア医の実践/視点」とは何なのでしょうか。仮にデータを、「このインタビュー調査に協力したプライマリ・ケア医の実践・視点」と限定するとしても、そうではない何かと比較がない限り、そう限定することも困難ではないでしょうか。
2. また本邦においては、プライマリ・ケア医の資格を持っていても何か特権的な裁量や義務が生じるわけではないと理解しています。また認定医を持っていなくともプライマリ・ケアを重要視する医師は多々います。このことを踏まえると、本論文がいうところの「プライマリ・ケア医の実践」とは、プライマリ・ケアの視点を大切にする医師の実践を指すのでしょうか。そうではなく「プライマリ・ケア認定医」を持つ医師の実践を指すのでしょうか。その辺りも判然としないところがあるため、もう少し明瞭にしていただきたく思います。

**【Major revision IV】スケーラブル/ノンスケーラブル概念は本論考において有効かつ必須の概念なのか**

 人工的には栽培できないマツタケ（ノンスケーラブル）が資本主義の市場の中に流通する（スケーラブル）様子を指すために導入された概念を、ワクチン接種の場に応用するのは少々概念の飛躍が過ぎ、かつチンが『マツタケ』においてこれら概念を用いて示したかったことともずれてしまうのではないでしょうか。

 そもそも標準医療の現場とは、個別性・偶発性（≒ノンスケーラブル）を重視する医師がいるとしても、国家資格、診療報酬制度、量産される薬剤や器材などに支えられたスケーラブルな現場です。言い換えると、標準医療はスケーラブルを前提とした現場であり、従ってそこでは、ワクチンに限らず全ての医療をスケーラブルにする努力と工夫は絶えずなされています。今回開発されたワクチンも製薬会社が大量生産したスケーラブルなものであり、その点で、チンの言う意味でのノンスケーラブルなマツタケとワクチンは相反するものであると言えないでしょうか。

 査読者はそう捉えるため、「スケーラブル」という概念を、チンが使ったマツタケの文脈とは大きく異なる標準医療の文脈に持ち込む意義がよくわかりませんでした。「新しいワクチンを速やかかつ大人数への接種するために、プライマリ・ケア医が行った工夫と努力の内実、およびその過程で生じた問題の数々を報告する」といった平易な表現では問題があるのでしょうか。

 「スケーラブル」という概念が本論文において有効かつ必須となる理由をもう少し説明してください。

**【Minor revision I】「ワクチン接種」という実践が指すところ**

 ここでいう「ワクチン接種」が何を指しているのかが今ひとつ明瞭でないと考えるため、もう少し整理をしてほしと思います。II-1に「ワクチン接種という実践に焦点を当てる研究はわずかである。確かに、1 回ごとの接種という行為は、医療に関わる行為の中で比較すれば高度な専門性が求められる技術だとはいえず 1、また時間としても 1 分足らずで終わってしまうため、「実践」として注目をひかないのかもしれない。」という記載がありますが、【結果】の箇所では、１分足らずで終わる実践だけではなく、ワクチンの準備や情報の周知についても言及がなされています。このため「実践」が指し示すところの意味がややぶれているように思えました。

（該当する場合は要回答）統計分析および解釈は適切ですか。

対象外（統計を使っていない）

本研究は明確かつ正確に提示されたものであり、最新の文献を引用していますか。

一部該当

方法と分析について第三者による再現が可能となるよう十分な詳細が提示されていますか。

いいえ

結果の基礎となるソースデータはすべて入手可能で再現性を十全に保証していますか。

一部該当

結論は結果により妥当な裏付けを得ていますか。

一部該当

研究設計は適切で学術的価値がありますか。

一部該当

Reviewer Expertise:

文化人類学、医療人類学

I confirm that I have read this submission and believe that I have an appropriate level of expertise to confirm that it is of an acceptable scientific standard, however I have significant reservations, as outlined above.

## References

[1] : Qualitative research: standards, challenges, and guidelines. *The Lancet* .2001;358(9280) : 10.1016/S0140-6736(01)05627-6 483-488 10.1016/S0140-6736(01)05627-6 11513933

# Response

**Published:** 2023-02-08

**Authors:** Shuhei Kimura

**Affiliations:** University of Tsukuba, Japan

このたびは大変お忙しい中、本稿の査読コメントをご執筆いただき、また本稿のインタビューデータおよび文化人類学者と医療者の協働という意義をご指摘いただき、どうもありがとうございました。

 以下、いただいた査読コメントに回答いたします。

**1.「人類学の方法」とは何か**

 II-2.方法 の箇所に「人類学の方法に基づく」「文化人類学の手法に基づいているため、厳密なコーディングを行っているわけではない」と記載されています。それではこの方法は一体いかなるものなのでしょうか。このことが不明瞭なまま書き進められていることは、（１）本稿がF1000Researchに投稿されたこと、（２）【２.方法】がCOREQを参照しながら書かれていること、（３）質的調査一般における本稿の位置付けの３点において問題であると思われます。

**【回答】**

 ご指摘ありがとうございます。まず、執筆者としては、f1000は多様な学問からの論文のプラットフォームですが、その論文の妥当性は、まずは当該分野（ないし近接領域）の専門家による査読を通じて判断されるものと理解しています。その意味で、本論文の立ち位置をより明確にするために、タイトルに「文化人類学的考察」を加え、冒頭のパラグラフでも「文化人類学の視点から」と付け加え、後述のように何をもって文化人類学とするかについても説明を加えました。また、「人類学」という言葉も、基本的には「文化人類学」に書き直しました（調査者のバックグラウンドで、文化／社会／医療など多様な場合は、そのまま「人類学」としています）。

 次にご指摘の２つの点ですが、「人類学の方法にもとづき、構造化しておらず（質問票は使っていない）、新型コロナウイルス感染症への対応や、現場から見える地域の様子、そしてその時々の話題（例えば2021年にはオリンピックやワクチン、変異株など）について、できるだけ自由に語ってもらうという形式をとった。」の部分は、具体的な内容については「もとづき」の後に書かれている通りです。ただここで「方法」と書いたのはミスリーディングだったかもしれません。「人類学の方法に基づき」を「 文化人類学における通常のフィールドワークの場合と同様」に修正します。また「自由に語ってもらう形式をとった」を、「 自由に語ってもらう ことで、できるだけインタビュアー側の想定によって内容を制限してしまうことなく、その時点におけるインタビュイーの関心事が浮かび上がるように心がけた」と加筆します。

 　もう一点も、ミスリーディングだったと思いますので、削除し、以下のように少し加筆します。

 本稿では、これまでに行った計75回のインタビューのうち、2021年２月から11月までに行った計25回のインタビューをデータとして用いた。まず、録画・録音したインタビューを文字起こしし、そこからワクチンに関わる部分を抽出し、各回を内容のまとまりごとに 切片化した（切片は最大 35 個になった）。そのうえで、内容の類似性と差異を比較しながら、10 のテーマ（接種の計画・オペレーション／接種の進捗／自施設の反応／自身について／患者の反応・患者への働きかけ／在宅患者への対応／反ワクチン／今後の展開・予想／ワクチンの効果）を見いだし 、それに沿って整理し、時系列に沿って大きな動きを把握するとともに、各時点でそれぞれの場所で何が問題となっていたのか、それにどのように対応したのか、傾向やパターン、および個々の特異性を分析するという作業を行った。 なお、文化人類学においては現地でのフィールドワークを行うことが中心だが、本稿ではパンデミックのため、フィールドワークは実施できなかった。しかし、上記のような姿勢でインタビューをすることでできるだけ対象者の視点に接近したこと、また既存の集合的カテゴリーや枠組みで対象者を理解するのではなく、対象者の言動のなかから現れてきた差異と共通性に即して集合的カテゴリーのあり方を、文化人類学の先行研究を手がかりにして考え直そうとする点で、文化人類学的な研究だと言える。

 もし、以上のやり方で、文化人類学の研究論文としてまだ問題があるようでしたら、ご指摘いただければ幸いです。

 また、（４）でご指摘の点ですが、タイトルにも「日本の」とあること、また地域性を考えてインタビュイーを選定していること、III章において日本におけるワクチン接種の大枠を説明し、そこにIV章のインタビュー結果を位置づけようとしていることで、日本という地域についての研究であることは明らかになっているかと考えます。ただ、V章において日本という言葉が明示されていなかったので、その点はややミスリーディングだったかと思いますので、第V章は、本稿の位置づけが明確になるよう、内容を変更せず、加筆しました。

**2. 外的妥当性と内的妥当性のいずれもが担保されていない**

 質的調査におけるデータの妥当性は、文化人類学の外側では常に議論される問題です。特に、本稿が文化人類学の外側の読者に向けられていると仮定すると、本論文におけるデータの外的妥当性・内的妥当性が共に担保されていないことはクリティカルな問題ではないでしょうか。

**【回答】**

 ご指摘ありがとうございます。まず結論は、論文である以上、当然、本論文で提示されたデータに基づく、執筆者の主張です。プライマリ・ケア医に特徴的な意見でも、調査者が重要だと思った個人の意見でもありません。もし第V章で書かれている内容が、本稿でそれまでに述べられてきた内容から飛躍があったり、矛盾している場合は、修正しなければならないと考えますので、具体的にご指摘いただければ幸いです。

 また内的／外的妥当性ですが、ご紹介いただいた論文では主にtransferabilityとして外的妥当性について論じられており、査読コメントもそちらに関わると思いますので、そちらについてお返事いたします。本稿の知見がどの範囲に当てはまるのかについての検討は、上記のように第V章でより明確に述べ、加えて第I章でプライマリ・ケア医を取り上げる理由について説明しました。それ以外に、①II章の２でインタビュイーの背景について説明していること、②III章で日本のワクチン接種の流れを説明したうえで、IV章の冒頭で日本のプライマリ・ケア医全体の中にインタビュイーたちを位置づけつつ、例えばIV章２節で「インタビュイーの偏り」について言及するなど、適宜一般化への留保を入れていること、③方法で述べた通り、取り上げる語りは１名だとしても、ある程度のパターンを見いだしたうえで全体を論じていること、などによって、どの程度の一般化が可能かについては示すことができていると考えています。

**3. 提示されたデータが「プライマリ・ケア医」の視点であることをどのように担保するのか/ 「プライマリ・ケア医」の視点とは何なのか**

 本邦で紹介されたプライマリ・ケア認定医へのインタビュー結果を、プライマリ・ケア認定医の視点や実践と呼ぶことについては留保が必要ではないでしょうか。例えば、本稿で言及がある、ワクチンへの疑念を示す人々に対しどのような説明をするか、所属施設においてワクチンを接種するスキームをどのように作るかは、プライマリ・ケア医に限られたものではなく、接種に関わった医療職全てが抱えた悩みと考えます。だとするならば、ここでいう「プライマリ・ケア医の実践/視点」とは何なのでしょうか。仮にデータを、「このインタビュー調査に協力したプライマリ・ケア医の実践・視点」と限定するとしても、そうではない何かと比較がない限り、そう限定することも困難ではないでしょうか。

 また本邦においては、プライマリ・ケア医の資格を持っていても何か特権的な裁量や義務が生じるわけではないと理解しています。また認定医を持っていなくともプライマリ・ケアを重要視する医師は多々います。このことを踏まえると、本論文がいうところの「プライマリ・ケア医の実践」とは、プライマリ・ケアの視点を大切にする医師の実践を指すのでしょうか。そうではなく「プライマリ・ケア認定医」を持つ医師の実践を指すのでしょうか。その辺りも判然としないところがあるため、もう少し明瞭にしていただきたく思います。

**【回答】**

 もちろん代表性について明確なことがいえないのは、文化人類学全般について常になされうる批判です。だからこそ、先のご指摘への回答で書いた通り、ワクチン接種に関わる仕組みでどのようなポジションを取っているかを説明し、それによって一般的（代表的）な働き方を示し、それとの関連の中に語りを位置づけており、インタビュイーの偏りについても留保を加え、また上述のように、I章に、本稿での「プライマリ・ケア医」が指すところについて加筆しました。

**4. スケーラブル/ノンスケーラブル概念は本論考において有効かつ必須の概念なのか**

 人工的には栽培できないマツタケ（ノンスケーラブル）が資本主義の市場の中に流通する（スケーラブル）様子を指すために導入された概念を、ワクチン接種の場に応用するのは少々概念の飛躍が過ぎ、かつチンが『マツタケ』においてこれら概念を用いて示したかったことともずれてしまうのではないでしょうか。

 そもそも標準医療の現場とは、個別性・偶発性（≒ノンスケーラブル）を重視する医師がいるとしても、国家資格、診療報酬制度、量産される薬剤や器材などに支えられたスケーラブルな現場です。言い換えると、標準医療はスケーラブルを前提とした現場であり、従ってそこでは、ワクチンに限らず全ての医療をスケーラブルにする努力と工夫は絶えずなされています。今回開発されたワクチンも製薬会社が大量生産したスケーラブルなものであり、その点で、チンの言う意味でのノンスケーラブルなマツタケとワクチンは相反するものであると言えないでしょうか。

 査読者はそう捉えるため、「スケーラブル」という概念を、チンが使ったマツタケの文脈とは大きく異なる標準医療の文脈に持ち込む意義がよくわかりませんでした。「新しいワクチンを速やかかつ大人数への接種するために、プライマリ・ケア医が行った工夫と努力の内実、およびその過程で生じた問題の数々を報告する」といった平易な表現では問題があるのでしょうか。

 「スケーラブル」という概念が本論文において有効かつ必須となる理由をもう少し説明してください。

**【回答】**

 ご指摘ありがとうございます。執筆者の理解では、チンのスケーラブル（資本主義だけでなく、国家、近代、産業化、人類の進歩、など多様なものに関わります）のイメージはむしろ彼女がこれまでフィールドとしていたインドネシアの大規模プランテーションにあると考えます。それがオレゴンの森林の話につながり、植林を計画したけれどもうまくいかなかった、というところでノンスケーラブルという言葉が出てきます。つまり、チンの議論では、「スケーラブルな計画／その実施において見いだされるノンスケーラビリティ」というように、両者はいわば対になって現れるものとして語られています。ご指摘の通り、医療やワクチン接種はスケーラブルを前提としているということは、本稿でも繰り返し説明している通りです。そのうえで、やはり実践のうえではそういかないこともある、という「事態」を、本稿ではノンスケーラブルと呼び、それを何とか「スケーラブルにしようとする」ものとして、今回の対象者たちの実践を取り上げています。その意味で、本稿がチンの論じ方から見て問題があると考えていません。

 本稿は、「プライマリ・ケア医たちが実際に行っていた雑多な事柄をどのようなものとしてまとめ、理解することができるのか」ということに対して、上記のプランテーションに比することのできる国家による大規模計画（国家とプランテーションの類比はジェームス・スコットも使っています）が現実的に生み出していた様々な問題への半ばバグ取り的なケアだったとして、スケーラブル／ノンスケーラブルという語彙を援用しています。

 なお付け加えておけば、チンは『マツタケ』で、ノンスケーラビリティを称揚し、スケーラビリティを批判する、というような立場を取っていないことも重要な点で（ノンスケーラブルなものも問題をもちうることもはっきり述べています）、執筆者も、本稿では明示していないですが、医療やワクチン接種に関して、スケーラビリティ／ノンスケーラビリティどちらも手放しで素晴らしいとも考えていません。ただ、プライマリ・ケア医たちが、何とかスケーラブルな計画が遂行できるよう、ノンスケーラブルな状況に対処していた、と述べています。

**5.「ワクチン接種」という実践が指すところ**

 ここでいう「ワクチン接種」が何を指しているのかが今ひとつ明瞭でないと考えるため、もう少し整理をしてほしと思います。II-1に「ワクチン接種という実践に焦点を当てる研究はわずかである。確かに、1 回ごとの接種という行為は、医療に関わる行為の中で比較すれば高度な専門性が求められる技術だとはいえず1、また時間としても 1 分足らずで終わってしまうため、「実践」として注目をひかないのかもしれない。」という記載がありますが、【結果】の箇所では、１分足らずで終わる実践だけではなく、ワクチンの準備や情報の周知についても言及がなされています。このため「実践」が指し示すところの意味がややぶれているように思えました。

**【回答】**

 ご指摘の箇所ですが、引用していただいているように、「行為」と「実践」という言葉を使い分けています。「ワクチン接種という実践」は、ワクチン接種に関わる準備や情報の周知などの様々な行為の総体という意味です。この点を第I章に加筆しました。
